# Genome-Wide Identification and Functional Analysis of Carboxylesterase and Methylesterase Gene Families in Peach (*Prunus persica* L. Batsch)

**DOI:** 10.3389/fpls.2019.01511

**Published:** 2019-11-22

**Authors:** Xiangmei Cao, Wenyi Duan, Chunyan Wei, Kunsong Chen, Don Grierson, Bo Zhang

**Affiliations:** ^1^Laboratory of Fruit Quality Biology/Zhejiang Provincial Key Laboratory of Horticultural Plant Integrative Biology, Zhejiang University, Hangzhou, China; ^2^Plant and Crop Sciences Division, School of Biosciences, University of Nottingham, Loughborough, Leicestershire, United Kingdom

**Keywords:** carboxylesterase, methylesterase, peach, volatile esters, methyl jasmonate, methyl salicylate

## Abstract

Carboxylesterases (CXE) and methylesterases (MES) are hydrolytic enzymes that act on carboxylic esters and are involved in plant metabolic processes and defense responses. A few functions of plant *CXE* and *MES* genes have been identified but very little information is available about the role of most members. We made a comprehensive study of this gene family in a commercially important species, peach (*Prunus persica* L. Batsch). A total of 33 peach *CXE* genes and 18 *MES* genes were identified and shown to be distributed unevenly between the chromosomes. Based on phylogenetic analysis, CXEs and MESs clustered into two different branches. Comparison of the positions of intron and differences in motifs revealed the evolutionary relationships between *CXE* and *MES* genes. RNA-seq revealed differential expression patterns of *CXE/MES*s in peach flower, leaf, and ripening fruit and in response to methyl jasmonate (MeJA) and ultraviolet B treatment. Transcript levels of candidate genes were verified by real-time quantitative PCR. Heterologous expression in *Escherichia coli* identified three CXEs that were involved in the hydrolysis of volatile esters *in vitro*. Furthermore, two recombinant MES proteins were identified that could hydrolyze MeJA and methyl salicylate. Our results provide an important resource for the identification of functional *CXE* and *MES* genes involved in the catabolism of volatile esters, responses to biotic and abiotic stresses and activation of signaling molecules such as MeJA and methyl salicylate.

## Introduction

Carboxylesterases (CXEs, EC 3.1.1.1) are ubiquitous enzymes which are present in all domains of life including some viruses. In the presence of water, CXEs catalyze the hydrolysis of a carboxylic ester to an alcohol and a carboxylic acid anion. However, compared to the broad ranges of functions established in mammals, insects, and microorganisms (Levisson et al., 2009; Wang et al., 2018; Wang et al., 2019), the biological roles of CXEs in plants are poorly understood. It is known that hydrolysis of natural compounds often alters their biological activity and transport and it has been suggested that CXEs play important roles in plants.

For classification, plant CXEs were divided into three classes in previous literature (Gershater and Edwards, 2007), class I mainly contains the previously annotated plant CXE family, including tobacco *hsr203J* and *AtCXE1-20* in *Arabidopsis* (Marshall et al., 2003); class II contains SA-binding protein (SABP2) from tobacco and methyl jasmonate esterase (MJE) from tomato; class III CXEs are related to the GDS lipases. In a later study, 20 genes with high sequence similarities to SABP2 were identified in *Arabidopsis* and phylogenetic analysis showed that they cluster into a clade that is distant from previously named *AtCXEs* (Yang et al., 2008). Due to their specific hydrolysis activity towards methyl jasmonate (MeJA), methyl salicylate (MeSA), and methyl indole-3-acetate (MeIAA), they were named the methylesterase (MES) family (Yang et al., 2008). For the sake of distinction and understanding, we name these two classes as CXE and MES families respectively, following the *Arabidopsis* nomenclature.

Structurally, CXE and MES both belong to the α/β hydrolase superfamily that contains the conserved catalytic triad of a serine (Ser), an aspartate (Asp) and a histidine (His) residue (Marshall et al., 2003; Yang et al., 2008). Functionally, MESs play important roles in plant defense responses and systemic acquired resistance. These enzymes have been systematically studied in *Arabidopsis*, where AtMES17 catalyzes the hydrolysis of MeIAA (Yang et al., 2008); AtMES2 catalyzes the hydrolysis of nicotinate methyl ester (MeNA) and is suppressed by abiotic stresses such as salt, abscisic acid, and mannitol (Wu et al., 2018); AtMES9 catalyzes the hydrolysis of MeSA and is significantly induced by pathogen infection (Vlot et al., 2008); AtMJE (AtMES10) converts MeJA to jasmonic acid (JA) and is significantly upregulated by MeJA treatment (Koo et al., 2013). In addition, grape MeJA can be hydrolyzed by VvMJE1, which is significantly upregulated by ultraviolet B (UV-B) treatment (Zhao et al., 2016). Compared to MESs, CXEs could hydrolyze a wider range of substrate esters containing different acyl groups such as straight chain (C1–C16), branched chain, or aryl esters (Cummins et al., 2007; Ileperuma et al., 2007). Therefore, CXEs are involved in plant growth, specialized metabolites synthesis, and plant defense responses (Winzer et al., 2012; Nomura et al., 2015; Schilmiller et al., 2016; Lin et al., 2017). CXE enzymes break down the waxy polymers of cutin present in the stigma cuticle, allowing the pollen tube to penetrate the stigma during germination (Hiscock et al., 2002; Rejón et al., 2012). Tobacco *hsr203J* is associated with detoxification of pathogen-derived compounds (Pontier et al., 1994). Induced expression of *CXE* genes in response to stresses has been observed in several plants (Gershater et al., 2007; Vlot et al., 2008; Islam and Yun, 2016). For example, *Arabidopsis AtCXE8* plays a role in promoting resistance to fungal invasion (Lee et al., 2013). Overexpressing a CXE (*PepEST)* resulted in enhanced resistance against an anthracnose fungus *Colletotrichum gloeosporioides* in transgenic pepper fruit (Ko et al., 2016). Volatile esters provide fruity-note aromas and contribute to the characteristic flavor of many fruits. The role of CXE proteins in regulating volatile ester content has been most extensively studied in tomato (*Solanum lycopersicum*), where SlCXE1 has a major role in determining volatile acetate ester content (Goulet et al., 2012). Apple fruit *MdCXE1* is associated with the hydrolysis of flavor esters such as butyl and hexyl acetate (Souleyre et al., 2011).

Peach (*Prunus persica* L. Batsch) is a member of the Rosaceae family and is one of the most popular fruit crops worldwide. For peach fruit, volatile acetate esters such as *Z*-3-hexenyl acetate are positively correlated with consumer preference (Bianchi et al., 2017). Besides their contribution to fruity notes, volatile esters also function as signal molecules in defense responses (Yamauchi et al., 2018; Hu et al., 2019). Reduced production of volatile esters resulted in hypersusceptibility to infection with *Pseudomonas syringae* pv. Tomato (López-Gresa et al., 2018) and a high content of peach *Z*-3-hexenyl acetate was associated with enhanced resistance to Mediterranean fruit fly *Ceratitis capitata* (Tabilio et al., 2013). Contents of peach fruit esters are also affected by ripening and postharvest storage treatment, including MeJA and UV-B (Zhang et al., 2010; Liu et al., 2017). As volatile esters, MeJA and MeSA are inactive mobile signaling molecules and need to be hydrolyzed to JA and SA to become active in plant defense responses.

Previous studies described above prompted us to investigate the potential functions of *CXE* and *MES* gene families in peach, which have not been studied previously. In the present study, genome-wide identification of *CXE* and *MES* was achieved by analysis of the peach genome database. Gene distribution on chromosomes, exon–intron architecture and differences in motifs were analyzed. In addition, patterns of transcript levels during fruit ripening, and in response to MeJA and UV-B treatments were investigated using an RNA-seq approach and verified by quantitative PCR (qPCR). Finally, enzymes active towards volatile esters or hormone derivatives were tested by using recombinant peach CXE and MES proteins produced in *Escherichia coli*.

## Materials and Methods

### Plant Materials and Treatments

Peach (*Prunus persica* L. Batsch cv. Hujingmilu) fruit at five different developmental stages, S1 (first rapid growth phase, 34 days after bloom, DAB), S2 (stone hardening, 71 DAB), S3 (second rapid growth phase, 94 DAB), S4 (mature stage,108 DAB), and S5 (ripening stage, 111DAB), were obtained from the Melting Peach Research Institute of Fenghua, Zhejiang Province, China (Wu et al., 2017). In the present study, peach fruits were subjected to three postharvest treatments. For ethylene treatment, fruits were placed in sealed buckets with 100 µl l^−1^ ethylene to accelerate ripening (Wu et al., 2019). Fruits sealed in air were used as controls. For MeJA treatment, fruits were soaked with 1 mM MeJA solution for 10 min, followed by storage at 20°C for 1 and 3 days (Qin et al., 2017). Peach fruits treated with distilled water were used as controls. Slices of flesh tissue (∼5 mm) were sampled. For UV-B treatment, fruits were exposed to 1.5 w m^−2^ for 6 h and 48 h at 20°C according to our previous study (Liu et al., 2017). Slices of peel (∼1 mm thick) were separated and immediately frozen in liquid nitrogen, then stored at −80° for further analysis. Three biological replicates with five fruits each were used at each sample time. Samples of flower and leaf were selected at the orchard, stored in liquid nitrogen, and transported to the laboratory.

### Identification of *CXE* and *MES* Genes and Chromosomal Map Construction

Peach *CXE* and *MES* genes were searched in the peach genome database at Phytozome (https://phytozome.jgi.doe.gov), based on gene annotation and confirmed by identifying the conserved GXSXG motif characteristic of CXE/MES members. The CXE and MES members in tobacco (*Nicotiana tabacum*), apple (*Malus domestica*), and grape (*Vitis vinifera*) were identified from the National Center for Biotechnology Information database. Tomato (*S. lycopersicum*) *CXE/MES* members were identified from Phytozome. *Arabidopsis CXE/MES* members were identified previously by Marshall et al. (2003) and Yang et al. (2008). Chromosome distribution of *CXE* and *MES* was generated by the MapChart (v2.3).

### Phylogenetic Analysis

Multiple sequence alignments of full-length predicted amino acid sequences were performed using Clustal W with default parameters [pairwise alignment, gap opening penalty: 10, gap extension penalty: 0.1, multiple alignment, gap opening penalty: 10, gap extension penalty: 0.2, protein weight matrix: Gonnet, residue-specific penalties: ON, hydrophilic penalties: ON, gap separation distance: 4, end gap separation: OFF, use negative matrix: OFF, delay divergent cutoff (%): 30). The phylogenetic analysis was constructed by the neighbor-joining method with 1,000 bootstrap replications by MEGA 6.0. The accession numbers of CXE and MES members of peach, *Arabidopsis*, tobacco, tomato, apple, and grape are listed in **Tables S1** and **S2**. The grouping of *CXE/MES* gene members in different species was based on the sequence similarity to the members in *Arabidopsis* (Marshall et al., 2003; Yang et al., 2008).

### Exon–Intron Organization and Motif Analysis

The exon–intron structure and intron phase information were obtained from the peach genome database. Intron phases were determined as follows: introns positioned between two codons were defined as phase 0, introns positioned between first and second base of codons were defined as phase 1, and introns positioned between second and third base were defined as phase 2. Exon–intron organization was visualized using the online Gene Structure Display Server 2.0. The conserved motifs were predicted by the online program MEME Suite (http://meme-suite.org/index.html).

### Analysis of the Promoters *cis*-Regulatory Elements

Putative promoter sequences (2 kb) of the peach genes were obtained from the peach genome database. Identification of potential *cis*-acting regulatory elements contained in the extracted promoter sequences was performed by Plant CARE (http://bioinformatics.psb.ugent.be/webtools/plantcare/html/).

### Gene Expression Analysis Using RNA-seq and qPCR

Total RNA was extracted according to Zhang et al. (2006), and quality was monitored by gel electrophoresis and A260/A280. Libraries for high-throughput Illumina strand-specific RNA-seq were prepared as described previously (Zhang et al., 2016). Three biological replicates for various samples were prepared. Real-time qPCR was used to verify the gene expression. qPCR was carried out with a Ssofast Eva Green Supermix Kit using a CFX96 instrument (Bio-Rad). The temperature program was as follows: 95°C for 3 min, 45 cycles of 95°C for 10 s, and 60°C for 30 s, with a final melting curve step from 65 to 95°C. Peach *TEF2* was used as an internal control to normalize small differences in template amounts (Zhang et al., 2010). The specificity of primers used for qPCR analysis was confirmed by product sequencing and are listed in **Table S3**.

### Volatiles Analysis by GC-MS

Volatiles were analyzed according to our previous study (Liu et al., 2017). Frozen fruits flesh (5 g) and peel (1 g) were ground into powder under liquid nitrogen and transferred to 20-ml vials containing 200 mM ethylenediaminetetraacetic acid and 20% CaCl_2_ solution. Before vials were sealed, 30 µl 2-octanol (0.8 mg ml^−1^) were added as internal standard. Volatiles were collected by a solid-phase microextraction fiber coated with 65 µm polydimethylsiloxane and divinylbenzene (Supelco Inc., USA). An Agilent 7890N gas chromatograph coupled with an Agilent 5975C mass spectrometer equipped with a DB-WAX column (0.32 mm, 30 m, 0.25 µm, J&W Scientific, Folsom, CA, USA) was used for volatiles identification. Carrier gas helium rate was 1.0 ml min^−1^. The temperature program started at 40°C and was increased by 3°C min^−1^ to 100°C and then to 245°C at 5°C min^−1^. The column effluent was ionized by electron ionization at an energy of 70 eV with a transfer temperature of 250°C and a source temperature of 230°C. Volatiles were identified by comparing their electron ionization mass spectra with the NIST Mass Spectral Library (NIST-08) and the retention time of authentic standards. Quantification of volatiles was performed using the peak area of the internal standard as a reference based on total ion chromatogram.

### Heterologous Protein Expression and Purification

The full-length coding sequences were cloned into pET-6×HN expression vector (Clontech, Mountain View, CA) with an N-terminal His-tag using primers in **Table S4**. The constructs were transformed into *E. coli* BL21(DE3) pLysS (Promega, Madison, WI, USA). The transformed cells were cultured at 37°C in Luria–Bertani medium until optical density at 600 nm reached 0.6. Isopropyl-β-D-thiogalactopyranoside was added to induce protein expression at 16°C overnight. The cells were collected by centrifugation (6,000 *g*, 4°C, 10 min), resuspended in Tris–HCl buffer (100 mM Tris, 2 mM dithiothreitol, pH 7.0), and were then disrupted by freeze–thawing. Recombinant proteins were purified by His TALON gravity column (Clontech) following the manufacturer's instructions. The presence of the recombinant proteins was confirmed by sodium dodecyl sulfate–polyacrylamide gel electrophoresis.

### Enzyme Activity Assay

Enzyme activity assays were performed in a 500-µl reaction mixture containing Tris–HCl buffer (50 mM Tris, pH 7.5, 2 mM dithiothreitol), 20 µl ester substrates (1 mM hexyl acetate, *E*-2-hexenyl acetate and *Z*-3-hexenyl acetate, butyl acetate, geranyl acetate, ethyl hexanoate, ethyl benzoate MeJA and MeSA, respectively) and 20 µl purified protein (1 µg µl^−1^). The reaction mixtures were incubated at 30°C for 30 min. The products of CXEs were detected by gas chromatography–mass spectrometry (GC-MS) as described above. For MESs activity analysis, the substrates (MeJA and MeSA) were detected by GC-MS as described above and the products (JA and SA) were extracted by 500 µl ethyl acetate. The extract solution was dried in vacuum condition, then redissolved with 100 µl methanol and identified by an Agilent 1290 Infinity LC System coupled with an Agilent 6460 Triple Quad mass spectrometer device (Agilent Technologies, USA) in negative ionization scan mode according to Wang et al. (2016). The scan range was 100–500 m/z. High-performance liquid chromatography analysis was performed using an Agilent Zorbax XDB C18 column (150 mm × 2.1 mm, 3.5 µm). The mobile phase consisted of a mixture of solvent A (0.1% formic acid in water) and solvent B (methanol) at a flowrate of 0.3 ml min^−1^ with the following gradient: 0–1.5 min, A/B at 60:40; followed by 6.5 min of solvent A/B at 0:100; subsequently returning to solvent A/B at 60:40 for 5 min until the end of the run.

### Chemicals and Reagents

The chemicals used for volatile identification and enzyme activity, including hexyl acetate, *E*-2-hexenyl acetate, *Z*-3- hexenyl acetate, hexanol, *E*-2-hexenol, *Z*-3-hexenol, 2-octanol, butyl acetate, geranyl acetate, ethyl hexanoate, ethyl benzoate, MeJA, MeSA, JA, and SA were purchased from Sigma-Aldrich (USA).

### Statistical Analysis

Heatmap figures were generated by Multi Experiment Viewer (version 4.6.0). The raw data of gene expression from RNA-seq used for heatmap analysis was listed in **Tables S5**–**S8**. The other figures were generated by Origin Pro 8 (OriginLab Corporation., Northampton, MA, USA). The two-sample significance test was calculated using unpaired Student's t-test (SPSS 19.0, SPSS Inc., Chicago, IL). Correlation analysis between transcript levels and ester contents was performed by MetaboAnalyst 4.0 (Chong et al., 2018).

### Accession Numbers

RNA sequencing raw sequence data of peach fruit samples generated from the present study can be found in the National Center for Biotechnology Information Short Read Archive database with accession number PRJNA576753 for peach samples at different development and ripening stages, PRJNA574777 for samples under ethylene treatment, PRJNA574004 for samples under MeJA treatment, and SRP103523 for samples under UV-B treatment.

## Results

### Genome-Wide Identification and Phylogenetic Analysis of CXE and MES Families

**Figure 1:**
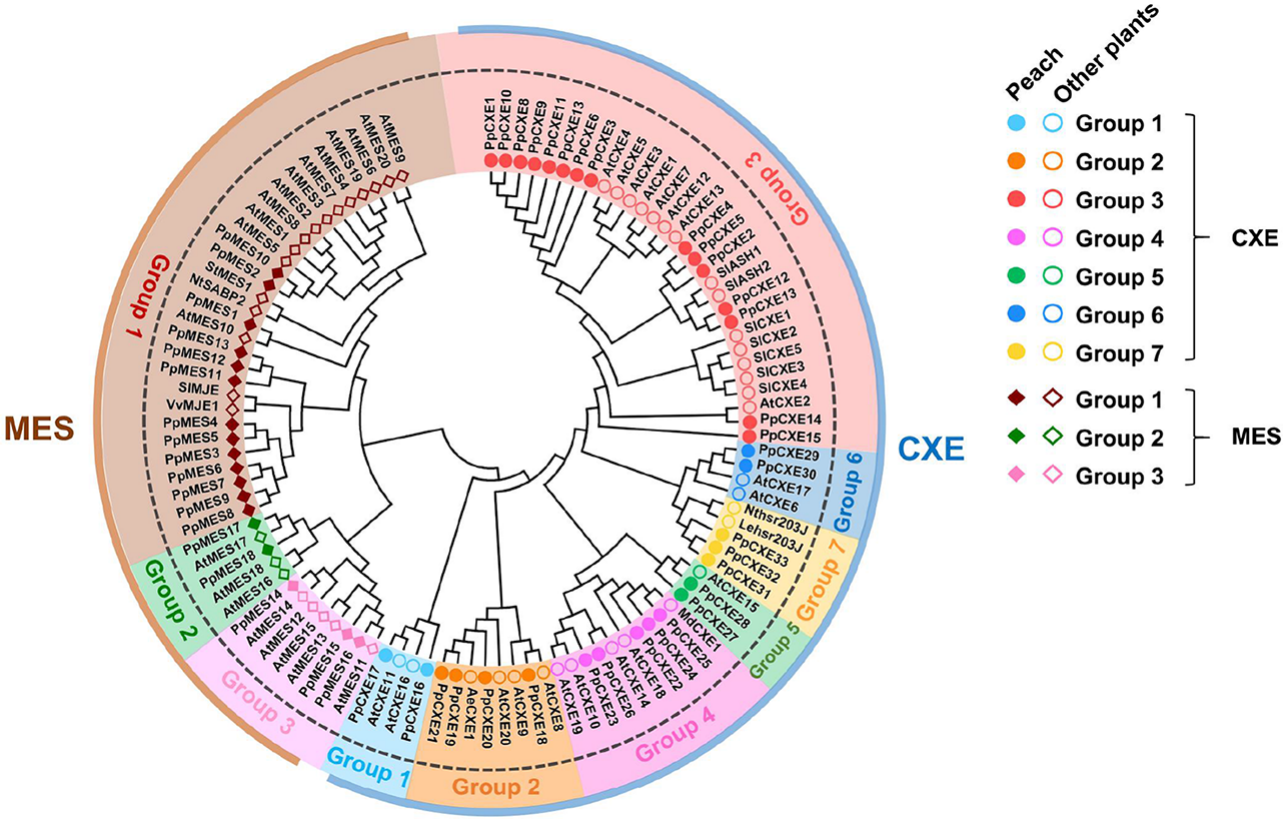
Phylogenetic analysis of CXE and MES families. The full-length amino acid sequences were aligned to construct the phylogenetic tree. The accession numbers of members are listed in **Tables S1** and **S2**. AeCXE1 (Q0ZPV7.1) from *Actinidia eriantha* and StMES1 (XP_006360243.1) from *Solanum tuberosum* are also included in the phylogenetic tree. The dots and circles represent CXEs from peach and other plants, respectively; filled and empty diamonds represent MESs from peach and other plants, respectively.

Based on gene annotation and the conserved GXSXG motif characteristic of CXE/MES members, a total of 33 peach CXE and 18 MES members were identified in the peach genome database. To reveal the phylogenetic relationships, a phylogenetic tree was generated using full-length deduced amino acid sequences from different plant species, including *Arabidopsis*, tomato, tobacco, and apple. The tree showed that MES and CXE members were clustered into two different classes (**Figure 1**). The CXE cluster includes AtCXE1-20 from *Arabidopsis* (Marshall et al., 2003) that could be further divided into seven groups, with group 3 with 15 peach members being the largest group (**Table 1**). Group 3 CXEs contained tomato SlCXE1 responsible for volatile esters hydrolysis (Goulet et al., 2012) and SlASH1 and SlASH2, which are responsible for the hydrolysis of acylsugars in tomato trichomes (Schilmiller et al., 2016). The MES cluster consisted of a further 20 *Arabidopsis* MESs (Yang et al., 2008), which were divided into three groups. The largest, group 1, contained 13 peach members (**Table 1**) and *Arabidopsis* AtMES10, which catalyzes the hydrolysis of MeJA (Koo et al., 2013). Tomato SlMJE and grape VvMJE1, associated with MeJA hydrolysis, were also clustered in group 1 (Stuhlfelder et al., 2004; Zhao et al., 2016). Moreover, tobacco NtSABP2, *Arabidopsis* AtMES4 and AtMES9 that are associated with MeSA metabolism (Forouhar et al., 2005; Vlot et al., 2008; Yang et al., 2008) were also observed in group 1 MESs. Group 2 includes AtMES17 and AtMES18, which are capable of hydrolyzing MeIAA (Yang et al., 2008).

**Table 1:** Number of the plant CXE and MES members in different groups.

Plant species	CXEs								MESs			
	Group 1	Group 2	Group 3	Group 4	Group 5	Group 6	Group 7	Total	Group 1	Group 2	Group 3	Total
Peach	2	4	15	5	2	2	3	**33**	13	2	3	**18**
*Arabidopsis*	2	3	8	4	1	2	0	**20** **^a^**	12	3	5	**20^b^**
Tobacco	6	9	9	2	4	4	7	**41**	12	4	12	**28**
Tomato	3	5	11	1	2	2	1	**25**	11	2	3	**16**
Apple	1	3	7	2	1	0	2	**16** **^c^**	12	2	5	**19**
Grape	1	9	11	7	4	8	3	**43**	11	1	3	**15^d^**

^a^Data from Marshall et al. (2003); ^b^data from Yang et al. (2008); ^c^data from Souleyre et al. (2011); ^d^data from Zhao et al. (2016). The grouping of CXE/MES gene members was based on the sequence similarity to the members in Arabidopsis. Gene IDs of CXE/MES gene members were listed in **Tables S1** and **S2**.

The distribution of plant cxes and mess is summarized in **Table 1**. In general, in *Arabidopsis*, tobacco, tomato, apple, and grape the largest number of cxes and mess were found in group 3 and group 1 respectively (**Table 1**), suggesting that these genes may play several important roles in plant metabolism.

### Chromosomal Locations of Peach *CXEs* and *MESs*

**Figure 2:**
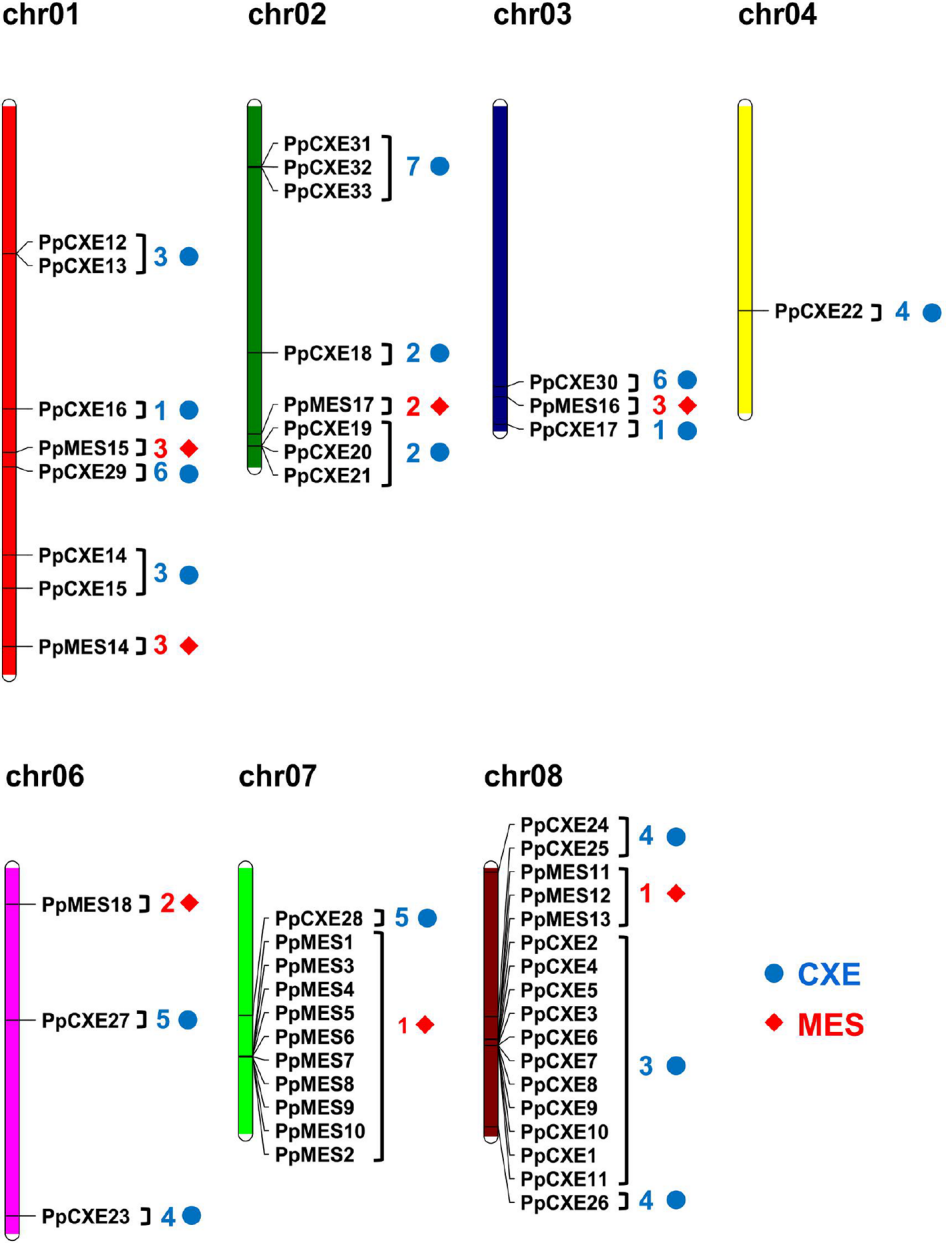
Chromosome distribution of peach *CXE* and *MES* genes. The chromosome numbers are shown at the top of each chromosome. *CXE* and *MES* are represented by blue dots and red diamonds respectively. Numbers next to gene names represent the phylogenetic group to which the genes belong. (e.g. 3 means group 3).

Chromosomal locations of the peach CXE and MES genes revealed that the genes were unevenly distributed (**Figure 2**). The largest gene cluster was observed on chromosome 08 (chr08), consisting of 17 genes. Eleven genes were located on chr07. Additionally, eight genes were found on each of chr01 and chr02. Only one gene was located on chr04 (**Figure 2**). For *CXEs*, genes from group 2 and group 7 were all located on chr02. Of the total 15 *CXEs* in group 3, 11 genes were located on chr08. Group 4 and group 5 *CXEs* were randomly distributed across four chromosomes (**Figure 2**). For *MESs*, group 1 consisting of 13 MESs had 10 members located on chr07 and 3 members on chr08. Group 2 MESs were located on chr02 and chr06. There were three members of group 3, with two located on chr01 and one on chr03 (**Figure 2**).

### Exon–Intron Structure and Motif Analysis of Peach *CXEs* and *MESs*

**Figure 3:**
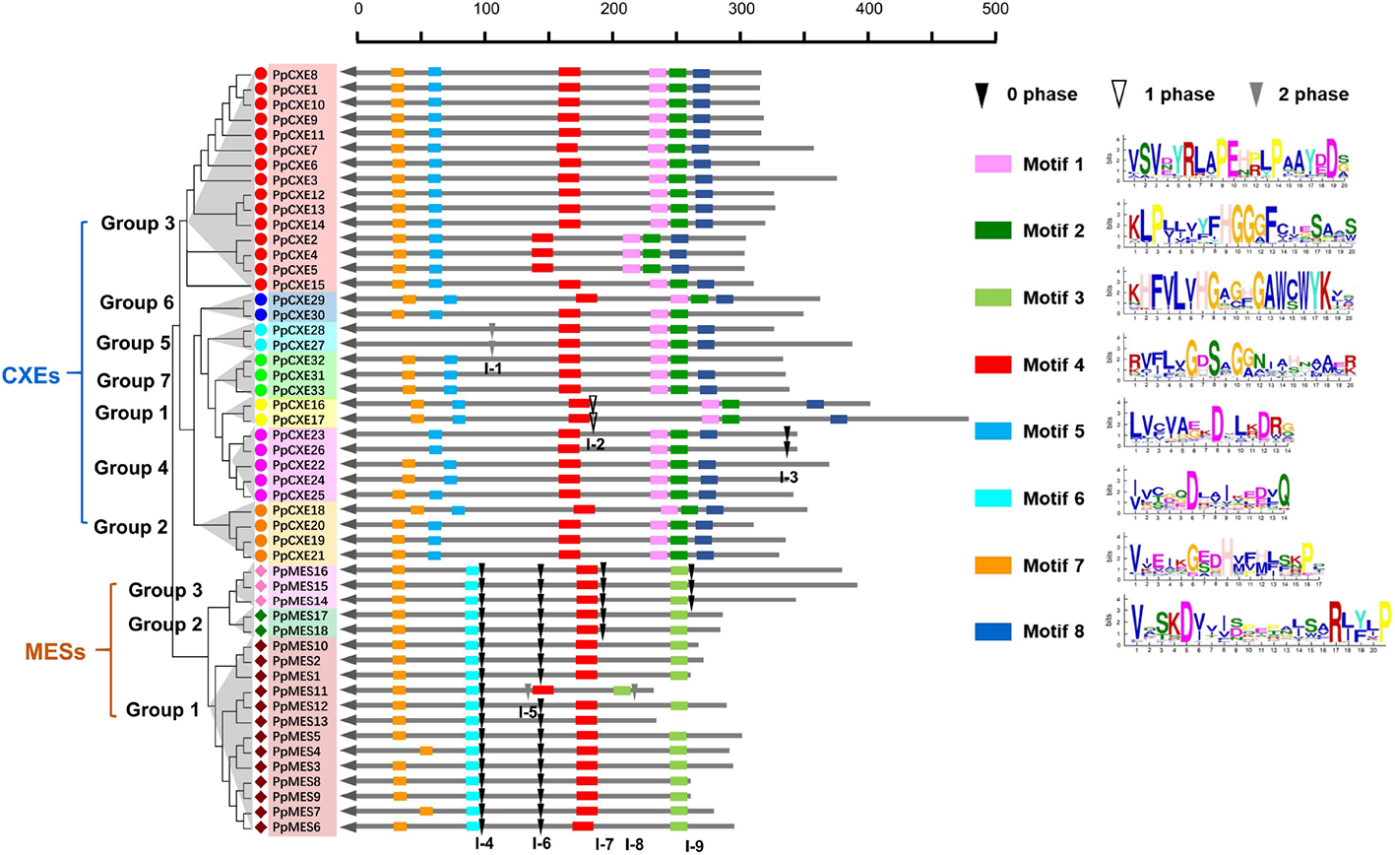
Distribution of introns and motif analysis of peach CXE and MES families. The introns were mapped and numbered according to the alignment of the amino acid sequences. The position of the arrow represents the C-terminal of the protein. The gray lines combined with the scale on the top indicates the length of the amino acid sequence. Inverted triangles indicate the position of introns. Intron phases 0, 1, and 2 are indicated by black inverted triangles, white inverted triangles, and gray inverted triangles, respectively. The rectangles in different colors on the right represent the different motifs with the consensus sequences shown alongside.

To investigate the evolutionary relationships within peach *CXE* and *MES* genes, the exon–intron structures were analyzed and it was found that *CXEs* had an entirely different intron distribution compared to *MESs.* Of the 33 peach *CXE* genes, 27 have no introns. A few introns were found in six *CXEs* in different phases (I-1, I-2, I-3) and were randomly distributed in different locations (**Figure 3**). In contrast, all MES members contain introns and at least four independent intron insertion events were observed. I-4 and I-6 are highly conserved in *MESs*, except for one member in group 1 (**Figure 3**). Intron I-7 is conserved in members of group 2 and 3 and I-9 in group 3 *MESs*. In addition, these four conserved introns are in the same phase (0 phase). The completely different intron insertions suggested the independent evolutionary relationship between CXE and MES family.

Using the online server MEME Suite, eight motifs were found in peach *CXEs* and *MESs* (**Figure 3**). Previous studies showed that both *CXE* and *MESs* contain the conserved catalytic triad, made up of a serine (Ser), an aspartate (Asp), and a histidine (His) residue (Marshall et al., 2003; Yang et al., 2008). All *CXE* and *MES* members contain the conserved GXSXG motif (motif 4) (**Figure 3**). Most *CXE* and *MES* members have motif 7, which contain the His residue. However, the Asp was located in different motifs, 5 and 6 respectively, in *CXE* and *MES*. An HGG motif (motif 2) found in *CXE* is believed to be a structural motif involved in stabilizing the substrate–enzyme intermediate during hydrolysis (Marshall et al., 2003; Ileperuma et al., 2007). However, the motif 3 in *MES* sequences was different, although the significance of this is not clear. Motif 1 and motif 8 were only present in *CXE* members (**Figure 3**), but their function is also not clear. These differences in motifs suggest functional differences between *CXE* and *MES* gene families.

### Analysis of the Promoter *cis*-Acting Regulatory Elements of *CXEs* and *MESs*

Abundant stress- and hormone-related *cis*-acting regulatory elements were detected in the regions 2 kb upstream of the transcription start site of all *CXE* and *MES* members (**Table S9**). Light-responsive *cis*-elements were identified in all the promoter regions analyzed and MYB-binding sites and MYC-binding sites were present in the promoters of almost all genes. Most *CXE* and *MES* promoters also contain abscisic acid and MeJA response elements. In addition, nine *CXE* members in group 3 and six *MES* members in group 1 also contain defense response *cis*-elements (**Table S9**).

### Expression Pattern of CXE and MES Genes in Vegetative and Reproductive Tissues

**Figure 4:**
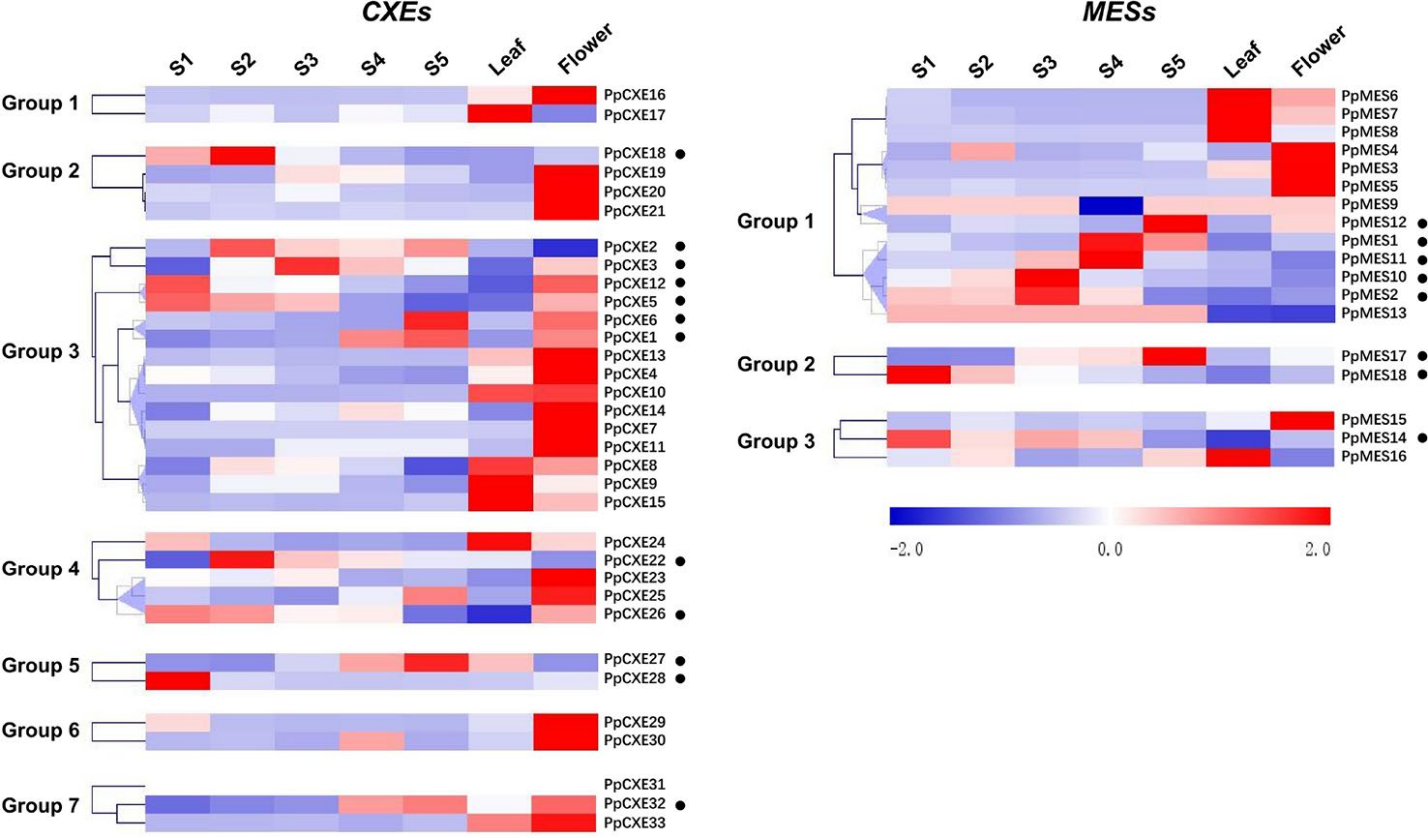
Expression pattern of peach *CXE* and *MES* genes in different tissues and during peach development and ripening. The expression patterns were analyzed by heatmap hierarchical clustering for leaf, flower, and fruit (S1–S5). Numbers on the left represent the phylogenetic groups. The black dots indicate the genes which have the highest transcript levels in fruit. The color scale is shown on the right; red represents high content, blue represents low content. Hierarchical clustering and heat map were constructed using MultiExperimentViewer (version 4.9.0).

To investigate the spatial and temporal distribution of *CXE* and *MES* transcripts in peach, expression patterns in flowers, leaves, and fruits at different developmental stages (S1–S5) were analyzed by RNA-seq. For *CXEs*, 15 members showed the highest transcript levels in flowers, five were predominately expressed in leaves, and 13 members were highly expressed in fruits (**Figure 4**). Transcripts of 6 out of 13 *CXEs* members were accumulated to high levels during ripening of peach fruits, including *PpCXE1*, *PpCXE2*, *PpCXE3*, and *PpCXE6* in group 3, *PpCXE27* in group 5, and *PpCXE32* in group 7 (**Figure 4**). Of the *MESs*, four were expressed in leaves and four in flowers and eight members were expressed mainly in peach fruits (**Figure 4**), where transcript of *PpMES1*, *PpMES11*, *PpMES12*, and *PpMES17* tended to accumulate during ripening (**Figure 4**).

### Gene Expression in Response to Ethylene, MeJA, and UV-B Treatments

**Figure 5:**
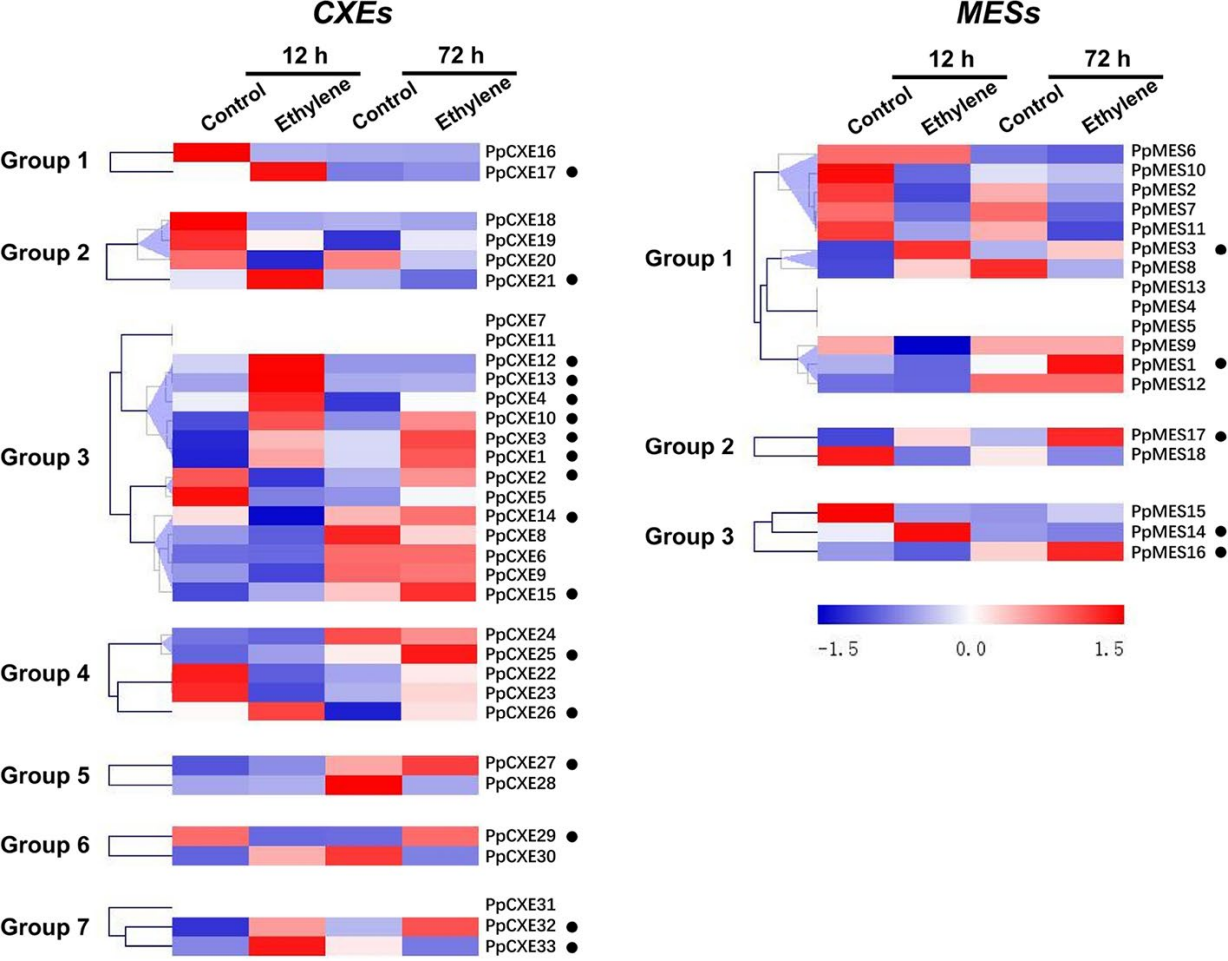
Expression pattern of peach *CXE* and *MES* genes in response to ethylene treatment. The expression pattern was analyzed by heatmap hierarchical clustering. 12 h and 72 h represent hours after fruit harvest. Numbers on the left represent the phylogenetic groups. The black dots indicate the genes whose transcript levels were upregulated by ethylene. The color scale is shown on the right; red represents high content, blue represents low content.

**Figure 6:**
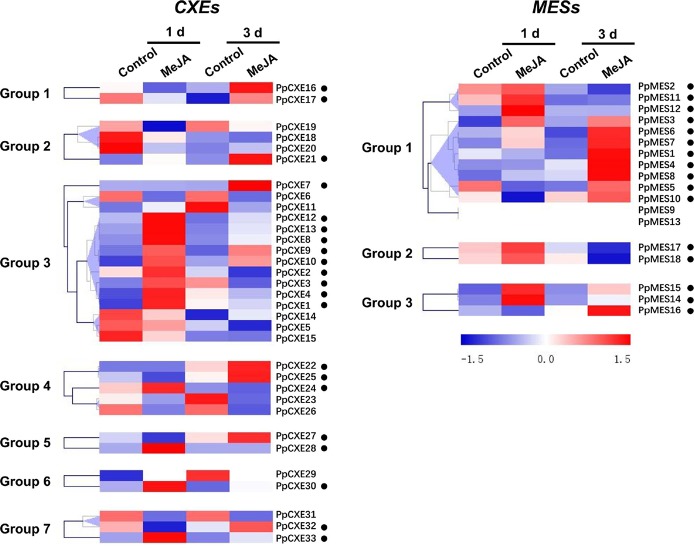
Expression pattern of peach *CXE* and *MES* genes in response to MeJA treatment. The expression pattern was analyzed by heatmap hierarchical clustering. 1 d and 3 d represent days after MeJA treatment. Numbers on the left represent the phylogenetic groups. The black dots indicate the genes whose transcript levels were up-regulated by MeJA. The color scale is shown on the right; red represents high content, blue represents low content.

To explore the physiological and functional relevance of peach *CXE* and *MES* genes, we analyzed the expression patterns in response to hormone and environmental stresses. For *CXEs*, accumulation of transcripts of 17 members was induced by ethylene treatment, including *PpCXE1* and *PpCXE3* from group 3 (**Figure 5** and **Figure S1**). Transcripts of five *MES* members were induced by ethylene, including *PpMES1* and *PpMES3* from group 1. Transcripts of 21 *CXEs* were induced by treatment with MeJA and the majority of *MES* members were also induced by MeJA including *PpMES1* and *PpMES3*. (**Figure 6** and **Figure S2**).

**Figure 7:**
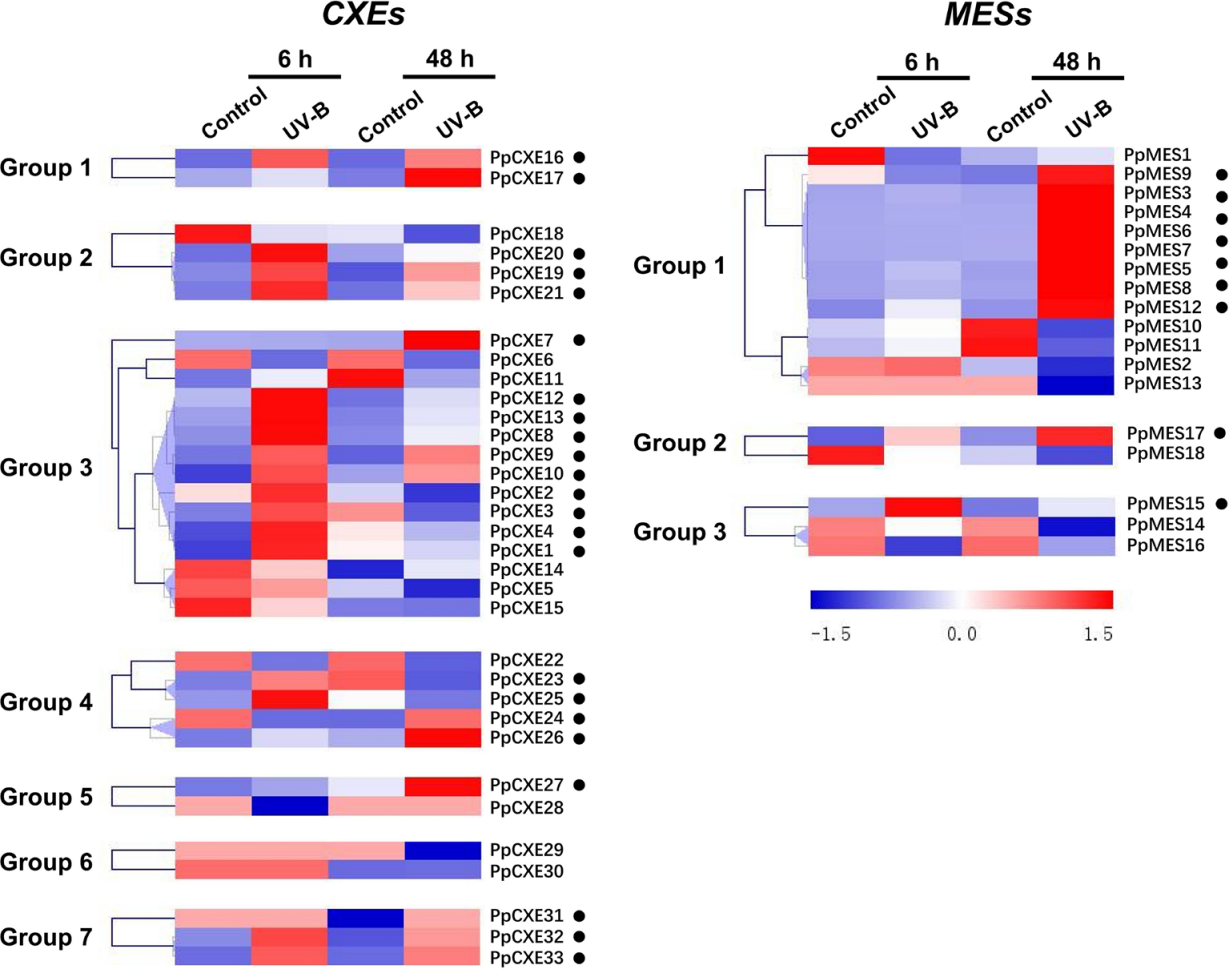
Expression pattern of peach *CXE* and *MES* genes in response to UV-B treatment. The expression pattern was analyzed by heatmap hierarchical clustering. 6 h and 8 h represent hours after UV-B treatment. Numbers on the left represent the phylogenetic groups. The black dots indicate the genes whose transcript levels were up-regulated by UV-B. The color scale is shown on the right; red represents high content, blue represents low content.

A total of 23 (62%) peach *CXE* genes were induced by UV-B irradiation, including 10 members in group 3 (**Figure 7**). After 48 h irradiation, transcripts of *PpCXE10* from group 3 were increased approximately 90-fold relative to the controls and significant increases in response to UV-B treatment was also observed for *PpCXE1* and *PpCXE8*, 7- and 20-fold respectively (**Figure S3**). For *MESs*, eight members in group 1 were induced by UV-B irradiation at 48 h, where transcript levels of *PpMES6* accumulated more than 70-fold (**Figure 7** and **Figure S3**). *PpMES17* in group 2 and *PpMES4* in group 3 were also induced by UV-B (**Figure 7**).

### Identification of CXEs Associated With Hydrolysis of Volatile Esters

**Figure 8:**
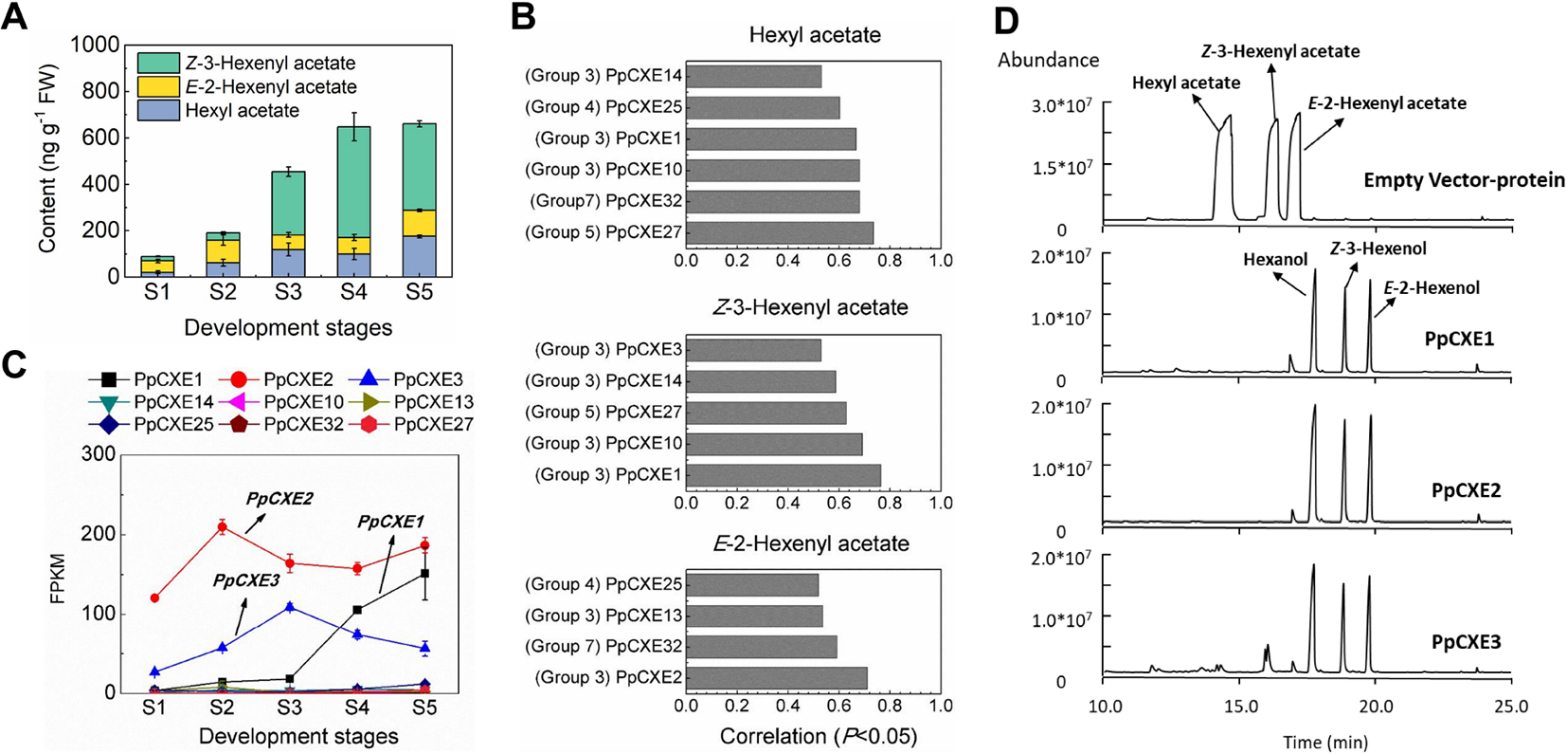
Correlation between *CXE* gene expression with content of volatile esters and the enzymatic activity of CXEs *in vitro*. **(A)** Content of volatile esters during fruit development and ripening. Error bars represent SE (*n* = 3). **(B)** Correlation between *CXE* genes expression with content of volatile esters (*P* < 0.05). **(C)** Transcript levels of *CXEs* during fruit ripening which showed significant positive correlation to volatile esters. Error bars represent SE (*n* = 3). **(D)** Enzymatic activity analysis of PpCXE1, PpCXE2, and PpCXE3 recombinant protein by GC-MS. Protein extracts from *E. coli* expressing empty vector was used as negative control.

Volatile esters contribute to the characteristic aroma of many fruits. For peach fruit, hexyl acetate, *E*-2-hexenyl acetate, and *Z*-3-hexenyl acetate are the main flavor-related esters (Zhang et al., 2010) that accumulated during ripening (**Figure 8A**). Expression of *CXE* members such as *SlCXE1* has been related to volatile esters metabolism in fruit (Goulet et al., 2012). To identify *CXE* genes associated with ester metabolism in peach, a correlation analysis between transcript levels and contents of volatile esters were carried out (**Figure 8B**). A total of nine *CXEs* were significantly positively correlated with volatile esters, six with hexyl acetate, five with *Z*-3-hexenyl acetate, and four with *E*-2-hexenyl acetate, respectively (**Figure 8B**). Three *CXEs* in group 3 exhibited relatively high transcript abundance throughout peach fruit ripening (**Figure 8C**). The highest transcript level in ripening fruit was observed for *PpCXE2* (*Prupe.8G120800*), followed by *PpCXE1* (*Prupe.8G121900*) and *PpCXE3* (*Prupe.8G121100*) (**Figure 8C** and **Figure S4**). Gene expression analysis by qPCR showed that *PpCXE2* had the highest transcript level, in agreement with the RNA-seq results. Moreover, *PpCXE1* expression increased approximately six-fold during fruit ripening (**Figure S4**) and *PpCXE1* and *PpCXE3* could be induced by ethylene, MeJA and UV-B (**Figures S5**–**S7**). These results showed that expression analysis of qPCR matched the pattern produced by RNA-seq. Therefore, these three CXEs were considered as candidates associated with ester metabolism and were selected for enzyme activity analysis.

Recombinant PpCXE1, PpCXE2, and PpCXE3 proteins purified from *E. coli* (**Figure S8**) were shown to hydrolyze the three esters (hexyl acetate, *E*-2-hexenyl acetate, *Z-*3-hexenyl acetate) to corresponding alcohols (**Figure 8D**). To explore CXE substrate specificity further, other esters with different lengths of acyl chains were also tested as substrates. PpCXE1 could hydrolyze acetates (C2) including butyl acetate and geraniol acetate to corresponding alcohols but showed no activity to ethyl hexanoate (C6) and ethyl benzoate (C7) (**Figure S9**). Similarly, it was not able to hydrolyze MeJA and MeSA (**Figure S9**).

### Identification of MESs Associated With Hydrolysis of MeJA and MeSA

**Figure 9:**
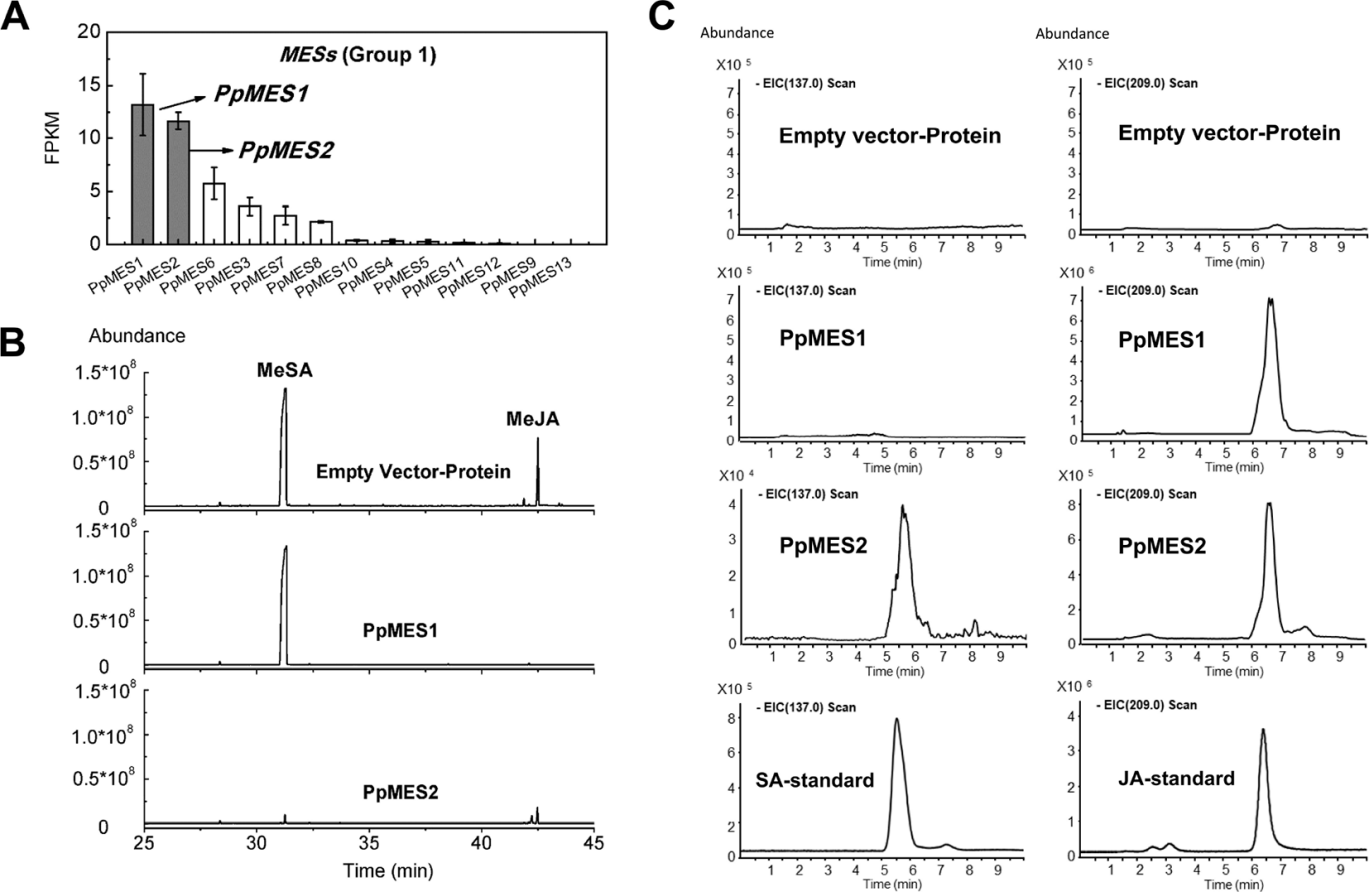
Transcript levels of *MES* genes and enzymatic activity of MESs *in vitro*. **(A)** Transcript levels of *MES* genes in group 1 in peach fruit. Error bars represent SE (*n* = 3). **(B)** Enzymatic activity of PpMES1 and PpMES2 recombinant protein by gas chromatography–mass spectrometry (GC-MS). MeSA and MeJA were used as substrates, and the products were detected by GC-MS after enzyme reaction. **(C)** Detection of recombinant protein reaction products by liquid chromatography–mass spectrometry (LC-MS). The scan fragment of SA and JA were 137 and 209 m/z, respectively. Protein extracts from *E. coli* expressing empty vector was used as negative control.

Based on phylogenetic analysis, group 1 MESs were clustered with members essential for hydrolysis of hormones, such as *Arabidopsis* AtMES10, tobacco NtSABP2, tomato SlMJE, and grape VvMJE1 (**Figure 1**). RNA-seq analysis revealed that *PpMES1* (*Prupe.7G140900*) and *PpMES2* (*Prupe.7G142200*) were the two members of the *MES* gene family with the highest transcript levels in peach fruit (**Figure 9A**). Moreover, qPCR showed transcripts of *PpMES1* increased five-fold during fruit ripening, and transcripts of *PpMES2* peaked at the S3 stage and decreased thereafter (**Figure S4**). Similar transcript accumulation patterns were observed comparing RNA-seq and qPCR. Furthermore, expression of *PpMES2* and *PpMES1* were induced by MeJA after 1 and 3 days treatment, respectively (**Figure 6**). Therefore, these two MESs members were selected for enzymatic activity analysis *in vitro*.

Recombinant protein PpMES1 could hydrolyze MeJA to JA but did not show activity toward MeSA (**Figures 9B, C**). Recombinant protein PpMES2 could hydrolyze MeSA to SA and MeJA to JA, respectively (**Figures 9B, C**). To further test if these two MESs could catalyze synthesis of other volatile esters, hexyl acetate, *Z-*3-hexenyl acetate, and *E*-2-hexenyl acetate were used as substrates for enzyme activity analysis. As shown in **Figure S10**, PpMES1 and PpMES2 were not able to hydrolyze hexyl acetate, *Z-*3-hexenyl acetate, and *E*-2-hexenyl acetate.

## Discussion

As members of the α/β superfamily of hydrolases, CXE and MES families catalyze a wide variety of hydrolytic reactions, typically the hydrolysis of ester bonds (Mindrebo et al., 2016). Functions of some CXE and MES members in *Arabidopsis*, tomato and other plants have been identified (Stuhlfelder et al., 2004; Yang et al., 2008; Goulet et al., 2012). Peach has been considered as a model plant of the Rosaceae family (Shulaev et al., 2008), however, knowledge of CXE and MES families remains unclear. In earlier studies, MESs was classified as a subclass of the CXE family (Gershater et al., 2007). Subsequent studies in *Arabidopsis* showed that the MES group is distant from the previously named AtCXEs cluster, according to the phylogenetic analysis (Yang et al., 2008). In this study, CXEs and MESs were compared using multiple approaches, including chromosome distribution, intron–exon structure, expression profiles, and catalytic activity of recombinant proteins.

Previous research has shown that events such as gain and loss of introns, as well as positions and phases of introns, are important cues for understanding evolution (Rogozin et al., 2000). Intron mapping revealed that most CXE members lacked introns compared to MESs and the different intron insertions indicated that they may have evolved independently. It is worth noting that introns I-4 and I-6 in MESs are conserved, I-7 is conserved in group 2 and group 3, and I-9 is conserved in group 3 (**Figure 3**), suggesting that the majority of conserved introns are ancient elements and their phases remain stable (Roy and Gilbert, 2005). Although both MESs and CXEs contain the conserved catalytic triad (Ser, Asp, and His) (Marshall et al., 2003; Li and Pu, 2016), the sequence contexts for this triad varies between the two gene types (**Figure 3**). There are also differences relating to motifs 1, 3, and 8 but the significance of this is not clear and their function needs further study.

Previous studies on a few CXEs have been performed in fruits due to the important physiological roles of volatile esters, which make an important contribution to the characteristic aroma of many ripening fruits. Sensory analysis indicated that volatile acetate esters such as *Z*-3-hexenyl acetate in peach fruit are positively correlated with consumer preference (Bianchi et al., 2017). The synthesis of volatile esters is catalyzed by alcohol acyltransferases (AATs), which transfer an acyl moiety from acyl-coenzyme A donors to alcohol acceptors. For example, tomato SlAAT1 and apple MdAAT1 were associated with volatile ester production during fruit ripening (Souleyre et al., 2014; Goulet et al., 2015). The role of CXE proteins in regulating volatile ester content has been most extensively studied in tomato (*S. lycopersicum*) where SlCXE1 hydrolyzed volatile esters (Goulet et al., 2012), determining volatile acetate ester content. Apple *MdCXE1* is also associated with hydrolysis of volatile esters (Souleyre et al., 2011). Here we show that the expression of nine *CXE* genes is significantly correlated with the increased patterns of the volatile esters during peach fruit ripening. The expression and role in hydrolysis of volatile esters of the three genes (*PpCXE1*, *PpCXE2*, and *PpCXE3*) with the highest transcript levels was confirmed by qPCR and overexpression in *E. coli* to determine their catalytic activity and substrate preferences. These results indicated that peach CXE genes are associated with catabolism of flavor-related volatile esters in peach fruit. Studies in tomato and apple showed that acyltransferases and CXE synergistically regulate the content of volatile esters in fruits (Souleyre et al., 2011; Goulet et al., 2012; Souleyre et al., 2014; Goulet et al., 2015), where increased transcript levels were observed for *AATs* and *CXEs* during fruit ripening.

Ethylene induced expression of some *CXE* and *MES* genes. RNA-seq and qPCR analysis revealed that ethylene could induce the transcript accumulation of *PpCXE1* and *PpCXE3*, which are involved in hydrolysis of esters. Ethylene is known to regulate fruit ripening and secondary metabolism (Giovannoni, 2007) and in previous studies, ethylene induced the expression of *AATs* and promoted the biosynthesis of volatile esters (Schaffer et al., 2007; Yang et al., 2016). *PpCXE1* in peach, *MdCXE1* in apple and *SlCXE1* in tomato all showed ripening-related expression patterns (Souleyre et al., 2011; Goulet et al., 2012). Similarly, *MdCXE16* in apple was also induced by ethylene (Souleyre et al., 2011). This suggests that ethylene response factors may be involved in regulating expression of these genes in fruits.

MES members are involved in hydrolysis of JA and SA conjugates. Hormone signaling molecules JA and SA are metabolized by methyltransferase to form inactive MeJA and MeSA, which are stored and transported in plants. MeJA and MeSA need to be hydrolyzed by MES in order to form JA and SA to activate the expression of downstream defense-related genes. Tomato SlMJE, *Arabidopsis* AtMJE (AtMES10), and grape VvMJE1 which could hydrolyze MeJA to JA (Stuhlfelder et al., 2004; Koo et al., 2013; Zhao et al., 2016). Tobacco SABP2 and *Arabidopsis* AtMES9 were associated with MeSA metabolism (Forouhar et al., 2005; Vlot et al., 2008; Yang et al., 2008). These members clustered in group 1, which contained a total of 13 peach *MESs*. RNA-seq revealed that *PpMES1* (*Prupe.7G140900*) and *PpMES2* (*Prupe.7G142200*) were the two gene family members with the most abundant transcripts in peach fruit. Moreover, expression of these two genes were significantly induced by MeJA treatment and this was confirmed by qPCR. As expected, recombinant *PpMES1* could use MeJA as a substrate for hydrolysis to produce JA *in vitro*. Both MeJA and MeSA could be hydrolyzed by recombinant *PpMES2* protein, yielding JA and SA, respectively. In addition to MeJA and MeSA, group 2 members in *Arabidopsis*, AtMES17 and AtMES18, could hydrolyze MeIAA to produce IAA (Yang et al., 2008). These results indicate that further analysis of *MES* genes would be an effective strategy for identifying enzymes with the ability to hydrolyze hormone conjugates and it will be interesting to test functions of group 2 peach *MES* members such as *PpMES17*and *PpMES18*.

RNA-seq showed that transcript levels of two CXEs (*PpCXE8* and *PpCXE10*) were induced significantly by MeJA and UV-B treatment. In addition, two MES family members *PpMES3* and *PpMES6* could be induced significantly by MeJA and UV-B (**Figures S2** and **S3**). The possibility that these members may play important roles in stress response needs further research.

## Conclusion

In summary, this study provides a fundamental framework and structural and functional information regarding the possible roles of CXE and MES families in peach. Based on phylogenetic analysis, a total of 51 peach members were divided into two classes, CXE and MES. RNA-seq analysis revealed tissue-specific expression in leaf, flower, and fruit and *CXE* and *MES* members affected by MeJA treatment and UV-B irradiation were identified. Heterologous expression in *E. coli* revealed that PpCXE1, PpCXE2, and PpCXE3 recombinant proteins are involved in hydrolysis of volatile esters important for ripening fruit quality. Furthermore, PpMES1 and PpMES2 recombinant proteins were shown to be associated with production of JA and SA through hydrolysis of MeJA and MeSA *in vitro*.

## Data Availability Statement

The relevant datasets can be found in the NCBI:

Samples at different development stages with accession number PRJNA576753 (https://www.ncbi.nlm.nih.gov/bioproject/PRJNA576753). Samples under ethylene treatment with accession number PRJNA574777 (https://www.ncbi.nlm.nih.gov/bioproject/PRJNA574777). Samples under MeJA treatment with accession number PRJNA574004 (https://www.ncbi.nlm.nih.gov/bioproject/PRJNA574004). Samples under UV-B treatment with accession number SRP103523 (https://trace.ncbi.nlm.nih.gov/Traces/study/?acc=SRP103523).

## Author Contributions

XC and BZ conceived the original screening and research plans. XC performed most of the experiments. WD performed postharvest MeJA treatment. CW performed the postharvest UV-B treatment. KC provided instruments for the experiments. DG provided guidance for experiments, assisted in data analysis and contributed to manuscript preparation. XC and BZ wrote the article with contributions of all the authors.

## Conflict of Interest

The authors declare that the research was conducted in the absence of any commercial or financial relationships that could be construed as a potential conflict of interest.

## References

[B1] Bianchi T. Weesepoel Y. Koot A. Iglesias I. Eduardo I. Gratacós-Cubarsí M. (2017). Investigation of the aroma of commercial peach (*Prunus persica* L. Batsch) types by Proton Transfer Reaction–Mass Spectrometry (PTR-MS) and sensory analysis. Food Res. Int. 99, 133–146. 10.1016/j.foodres.2017.05.007 28784469

[B2] Chong J. Soufan O. Li C. Caraus L. Li S. Bourque G. (2018). MetaboAnalyst 4.0: towards more transparent and integrative metabolomics analysis. Nucleic Acids Res. 46, W486–W494. 10.1093/nar/gky310 29762782 PMC6030889

[B3] Cummins I. Landrum M. Steel P. G. Edwards R. (2007). Structure activity studies with xenobiotic substrates using carboxylesterases isolated from Arabidopsis thaliana. Phytochem. 68, 811–818. 10.1016/j.phytochem.2006.12.014 17270225

[B4] Forouhar F. Yang Y. Kumar D. Chen Y. Fridman E. Park S. W. (2005). Structural and biochemical studies identify tobacco SABP2 as a methyl salicylate esterase and implicate it in plant innate immunity. Proc. Natl. Acad. Sci. U. S. A. 102, 1773–1778. 10.1073/pnas.0409227102 15668381 PMC547883

[B5] Gershater M. C. Edwards R. (2007). Regulating biological activity in plants with carboxylesterases. Plant Sci. 173, 579–598. 10.1016/j.plantsci.2007.08.008

[B6] Gershater M. C. Cummins I. Edwards R. (2007). Role of a carboxylesterase in herbicide bioactivation in *Arabidopsis thaliana* . J. Biol. Chem. 282, 21460–21466. 10.1074/jbc.M701985200 17519238

[B7] Giovannoni J. J. (2007). Fruit ripening mutants yield insights into ripening control. Curr. Opin. Plant Biol. 10, 283–289. 10.1016/j.pbi.2007.04.008 17442612

[B8] Goulet C. Mageroy M. H. Lam N. B. Floystad A. Tieman D. M. Klee H. J. (2012). Role of an esterase in flavor volatile variation within the tomato clade. Proc. Natl. Acad. Sci. U. S. A. 109, 19009–19014. 10.1073/pnas.1216515109 23112200 PMC3503167

[B9] Goulet C. Kamiyoshihara Y. Lam N. B. Richard T. Taylor M. G. Tieman D. M. (2015). Divergence in the enzymatic activities of a Tomato and *Solanum pennellii* alcohol acyltransferase impacts fruit volatile ester composition. Mol. Plant 8, 153–162. 10.1016/j.molp.2014.11.007 25578279

[B10] Hiscock S. J. Bown D. Gurr S. J. Dickinson H. G. (2002). Serine esterases are required for pollen tube penetration of the stigma in Brassica. Sex Plant Reprod. 15, 65–74. 10.1007/s00497-002-0143-7

[B11] Hu L. Ye M. Erb M. (2019). Integration of two herbivore-induced plant volatiles results in synergistic effects on plant defence and resistance. Plant Cell Environ. 42, 959–971. 10.1111/pce.13443 30195252 PMC6392123

[B12] Ileperuma N. R. Marshall S. D. Squire C. J. Baker H. M. Oakeshott J. G. Russell R. J. (2007). High-resolution crystal structure of plant carboxylesterase AeCXE1, from *Actinidia eriantha*, and its complex with a high-affinity inhibitor paraoxon. Biochemistry 46, 1851–1859. 10.1021/bi062046w 17256879

[B13] Islam M. Z. Yun H. K. (2016). Identification and expression profiles of six transcripts encoding carboxylesterase protein in Vitis flexuosa infected with pathogens. Plant Pathol. J. 32, 347–356. 10.5423/PPJ.OA.11.2015.0241 27493610 PMC4968645

[B14] Ko M. Cho J. H. Seo H. H. Lee H. H. Kang H. Y. Nguyen T. S. (2016). Constitutive expression of a fungus-inducible carboxylesterase improves disease resistance in transgenic pepper plants. Planta 244, 379–392. 10.1007/s00425-016-2514-6 27074836

[B15] Koo Y. J. Yoon E. S. Seo J. S. Kim J. K. Choi Y. D. (2013). Characterization of a methyl jasmonate specific esterase in Arabidopsis. J. Korean Soc Appl. Biol. Chem. 56, 27–33. 10.1007/s13765-012-2201-7

[B16] López-Gresa M. P. Payá C. Ozáez M. Rodrigo I. Conejero V. Klee H. (2018). A new role for green leaf volatile esters in tomato stomatal defense against *Pseudomonas syringe* pv. *tomato* . Front. Plant Sci. 9, 1855. 10.3389/fpls.2018.01855 30619420 PMC6305539

[B17] Lee S. Hwang S. Seo Y. W. Jeon W. B. Oh B. J. (2013). Molecular characterization of the AtCXE8 gene, which promotes resistance to Botrytis cinerea infection. Plant Biotechnol. Rep. 7, 109–119. 10.1007/s11816-012-0253-0

[B18] Levisson M. van der Oost J. Kengen S. W. (2009). Carboxylic ester hydrolases from hyperthermophiles. Extremophiles 13, 567–581. 10.1007/s00792-009-0260-4 19544040 PMC2706381

[B19] Li H. Pu H. (2016). Crystal structure of methylesterase family member 16 (MES16) from *Arabidopsis thaliana* . Biochem. Biophys. Res. Commun. 474, 226–231. 10.1016/j.bbrc.2016.04.115 27109476

[B20] Lin Q. Chen S. Chao Y. Huang X. Wang S. Qiu R. (2017). Carboxylesterase-involved metabolism of di-n-butyl phthalate in pumpkin (*Cucurbita moschata*) seedlings. Environ. Pollut. 220, 421–430. 10.1016/j.envpol.2016.09.084 27697378

[B21] Liu H. Cao X. Liu X. Xin R. Wang J. J. Gao J. (2017). UV-B irradiation differentially regulates terpene synthases and terpene content of peach. Plant Cell Environ. 40, 2261–2275. 10.1111/pce.13029 28722114

[B22] Marshall S. D. Putterill J. J. Plummer K. M. Newcomb R. D. (2003). The carboxylesterase gene family from *Arabidopsis thaliana* . J. Mol. Evol. 57, 487–500. 10.1007/s00239-003-2492-8 14738307

[B23] Mindrebo J. T. Nartey C. M. Seto Y. Burkart M. D. Noel J. P. (2016). Unveiling the functional diversity of the alpha/beta hydrolase superfamily in the plant kingdom. Curr. Opin. Struct. Biol. 41, 233–246. /101016/j.sbi.201608.005 27662376 10.1016/j.sbi.2016.08.005 PMC5687975

[B24] Nomura T. Murase T. Ogita S. Kato Y. (2015). Molecular identification of tuliposide B-converting enzyme: a lactone-forming carboxylesterase from the pollen of tulip. Plant J. 83, 252–262. 10.1111/tpj.12883 25997073

[B25] Pontier D. Godiard L. Marco Y. Roby D. (1994). hsr203J, a tobacco gene whose activation is rapid, highly localized and specific for incompatible plant/pathogen interactions. Plant J. 5, 507–521. 10.1046/j.1365-313X.1994.5040507.x 8012404

[B26] Qin G. H. Wei S. W. Tao S. T. Zhang H. P. Huang W. J. Yao G. F. (2017). Effects of postharvest methyl jasmonate treatment on aromatic volatile biosynthesis by 'Nanguoli' fruit at different harvest maturity stages. N. Z. J. Crop Hortic. Sci. 45, 191–201. 10.1080/01140671.2016.1272470

[B27] Rejón J. D. Zienkiweicz A. Rodríguez-García M. Castro A. J. (2012). Profiling and functional classification of esterases in olive (*Olea europaea*) pollen during germination. Ann. Bot. 110, 1035–1045. 10.1093/aob/mcs174 22922586 PMC3448428

[B28] Rogozin I. B. Lyons-Weiler J. Koonin E. V. (2000). Intron sliding in conserved gene families. Trends Genet. 16, 430–432. S0168-9525(00)02096-5 11050324 10.1016/s0168-9525(00)02096-5

[B29] Roy S. W. Gilbert W. (2005). Rates of intron loss and gain: implications for early eukaryotic evolution. Proc. Natl. Acad. Sci. U.S.A. 102, 5773–5778. 10.1073/pnas.0500383102 15827119 PMC556292

[B30] Schaffer R. J. Friel E. N. Souleyre E. J. Bolitho K. Thodey K. Ledger S. (2007). A genomics approach reveals that aroma production in apple is controlled by ethylene predominantly at the final step in each biosynthetic pathway. Plant Physiol. 144, 1899–1912. 10.1104/pp.106.093765 17556515 PMC1949883

[B31] Schilmiller A. L. Gilgallon K. Ghosh B. Jones A. D. Last R. L. (2016). Acylsugar acylhydrolyases: carboxylesterase-catalyzed hydrolysis of acylsugars in tomato trichomes. Plant Physiol. 170, 1331–1344. 10.1104/pp.15.01348 26811191 PMC4775116

[B32] Shulaev V. Korban S. S. Sosinski B. Abbott A. G. Aldwinckle H. S. Folta K. M. (2008). Multiple models for Rosaceae genomics. Plant Physiol. 147, 985–1003. 10.1104/pp.107.115618 18487361 PMC2442536

[B33] Souleyre E. J. Marshall S. D. Oakeshott J. G. Russell R. J. Plummer K. M. Newcomb R. D. (2011). Biochemical characterisation of MdCXE1, a carboxylesterase from apple that is expressed during fruit ripening. Phytochemistry 72, 564–571. 10.1016/j.phytochem.2011.01.020 21315388

[B34] Souleyre E. J. Chagne D. Chen X. Tomes S. Turner R. M. Wang M. Y. (2014). The AAT1 locus is critical for the biosynthesis of esters contributing to 'ripe apple' flavour in 'Royal Gala' and 'Granny Smith' apples. Plant J. 78, 903–915. 10.1111/tpj.12518 24661745

[B35] Stuhlfelder C. Mueller M. J. Warzecha H. (2004). Cloning and expression of a tomato cDNA encoding a methyl jasmonate cleaving esterase. Eur. J. Biochem. 271, 2976–2983. 10.1111/j.1432-1033.2004.04227.x 15233793

[B36] Tabilio M. R. Fiorini D. Marcantoni E. Materazzi S. Delfini M. De Salvador F. R. (2013). Impact of the Mediterranean fruit fly (medfly) *Ceratitis capitata* on different peach cultivars: the possible role of peach volatile compounds. Food Chem. 140, 375–381. 10.1016/j.foodchem.2013.02.074 23578656

[B37] Vlot A. C. Liu P. P. Cameron R. K. Park S. W. Yang Y. Kumar D. (2008). Identification of likely orthologs of tobacco salicylic acid-binding protein 2 and their role in systemic acquired resistance in *Arabidopsis thaliana*. Plant J. 56, 445–456. 10.1111/j.1365-313X.2008.03618.x 18643994

[B38] Wang F. Guo Z. Li H. Wang M. Onac E. Zhou J. (2016). Phytochrome A and B function antagonistically to regulate cold tolerance *via* abscisic acid-dependent jasmonate signaling. Plant Physiol. 170, 459–471. 10.1104/pp.15.01171 26527654 PMC4704577

[B39] Wang K. Huang Y. Li X. Chen M. (2018). Functional analysis of a carboxylesterase gene associated with isoprocarb and cyhalothrin resistance in *Rhopalosiphum padi* (L.). Front. Physiol. 9, 992. 10.3389/fphys.2018.00992 30090072 PMC6068260

[B40] Wang X. Shi J. Zhu H. J. (2019). Functional study of carboxylesterase 1 protein isoforms. Proteomics 19, 1800288. 10.1002/pmic.201800288 PMC6377296 30520264

[B41] Winzer T. Gazda V. He Z. Kaminski F. Kern M. Larson T. R. (2012). A *Papaver somniferum* 10-gene cluster for synthesis of the anticancer alkaloid noscapine. Science 336, 1704–1708. 10.1126/science.1220757 22653730

[B42] Wu B. Gao L. Gao L. Xu Y. Liu H. Cao X. (2017). Genome-wide identification, expression patterns, and functional analysis of UDP glycosyltransferase family in peach (*Prunus persica* L. Batsch). Front. Plant Sci. 8, 389. 10.3389/fpls.2017.00389 28382047 PMC5360731

[B43] Wu R. Zhang F. Liu L. Li W. Pichersky E. Wang G. (2018). MeNA, controlled by reversible methylation of nicotinate, is an NAD precursor that undergoes long-distance transport in Arabidopsis. Mol. Plant 11, 1264–1277. 10.1016/j.molp.2018.07.003 30055263

[B44] Wu B. Cao X. Liu H. Zhu C. Klee H. Zhang B. (2019). UDP-glucosyltransferase PpUGT85A2 controls volatile glycosylation in peach. J. Exp. Bot. 70, 925–936. 10.1093/jxb/ery419 30481327 PMC6363097

[B45] Yamauchi Y. Matsuda A. Matsuura N. Mizutani M. Sugimoto Y. (2018). Transcriptome analysis of *Arabidopsis thaliana* treated with green leaf volatiles: possible role of green leaf volatiles as self-made damage-associated molecular patterns. J. Pestic. Sci. 43, 207–213. 10.1584/jpestics.D18-020 30363142 PMC6140709

[B46] Yang Y. Xu R. Ma C. J. Vlot A. C. Klessig D. F. Pichersky E. (2008). Inactive methyl indole-3-acetic acid ester can be hydrolyzed and activated by several esterases belonging to the AtMES esterase family of Arabidopsis. Plant Physiol. 147, 1034–1045. 10.1104/pp.108.118224 18467465 PMC2442527

[B47] Yang X. Song J. Du L. Forney C. Leslie C. P. Sherry F. (2016). Ethylene and 1-MCP regulate major volatile biosynthetic pathways in apple fruit. Food Chem. 194, 325–336. 10.1016/j.foodchem.2015.08.018 26471562

[B48] Zhang B. Chen K. Bowen J. Allan A. Espley R. Karunairetnam S. (2006). Differential expression within the LOX gene family in ripening kiwifruit. J. Exp. Bot. 57, 3825–3836. 10.1093/jxb/erl151 17032731

[B49] Zhang B. Shen J. Y. Wei W. W. Xi W. P. Xu C. J. Ferguson I. (2010).Expression of genes associated with aroma formation derived from the fatty acid pathway during peach fruit ripening. J. Agric. Food Chem. 58, 6157–6165. 10.1021/jf100172e 20415420

[B50] Zhang B. Tieman D. M. Jiao C. Xu Y. Chen K. Fei Z. (2016). Chilling-induced tomato flavor loss is associated with altered volatile synthesis and transient changes in DNA methylation. Proc. Natl. Acad. Sci. U. S. A. 113, 12580–12585. /10.1073/pnas.1613910113 27791156 10.1073/pnas.1613910113 PMC5098663

[B51] Zhao N. Lin H. Lan S. Jia Q. Chen X. Guo H. (2016). VvMJE1 of the grapevine (*Vitis vinifera*) VvMES methylesterase family encodes for methyl jasmonate esterase and has a role in stress response. Plant Physiol. Biochem. 102, 125–132. 10.1016/j.plaphy.2016.02.027 26934101

