# Supplementary material for: Genome-Wide Identification and Functional Analysis of Carboxylesterase and Methylesterase Gene Families in Peach (Prunus persica L. Batsch)
Source: Front Plant Sci. 2019 Nov 22;10:1511. doi: 10.3389/fpls.2019.01511 (PMC6884059; doi:10.3389/fpls.2019.01511)
Supplement: Supplementary file 1 [file DataSheet_1.docx]

Supplementary Material

**Supplementary Table S1.** CXE members in peach, Arabidopsis, tobacco, tomato, apple and grape.

| Groups | Peach | Arabidopsis | Tobacco | Tomato | Apple | Grape |
| --- | --- | --- | --- | --- | --- | --- |
| Group 1 | Prupe.1G237400 (PpCXE16) | At3g27320 (AtCXE11) | XP_016434975.1 | Solyc05g014320 | EB106571 (MdCXE16) | XP_002267088.1 |
|  | Prupe.3G299300 (PpCXE17) | At5g14310 (AtCXE16) | XP_016480836.1 | Solyc03g005100 |  |  |
|  |  |  | XP_016502349.1 | Solyc02g093790 |  |  |
|  |  |  | XP_016500645.1 |  |  |  |
|  |  |  | XP_016497504.1 |  |  |  |
|  |  |  | XP_016449258.1 |  |  |  |
| Group 2 | Prupe.2G149600 (PpCXE18) | At2g45600 (AtCXE8) | XP_016514594.1 | Solyc01g094010 | ES789967 (MdCXE4) | XP_019081511.1 |
|  | Prupe.2G286500 (PpCXE19) | At2g45610 (AtCXE9) | XP_016473898.1 | Solyc01g098140 | EG631362 (MdCXE14) | XP_010662449.1 |
|  | Prupe.2G286600 (PpCXE20) | At5g62180 (AtCXE20) | XP_016455878.1 | Solyc07g040890 | CN942679 (MdCXE15) | XP_003633918.1 |
|  | Prupe.2G286700 (PpCXE21) |  | XP_016454448.1 | Solyc06g035520 |  | XP_002277119.1 |
|  |  |  | XP_016454162.1 | Solyc01g091410 |  | XP_002277011.2 |
|  |  |  | XP_016442088.1 |  |  | XP_002272186.4 |
|  |  |  | XP_016436196.1 |  |  | XP_002271453.2 |
|  |  |  | XP_016511474.1 |  |  | XP_002267605.1 |
|  |  |  | XP_016478801.1 |  |  | XP_002267455.1 |
| Group 3 | Prupe.8G121900 (PpCXE1) | At1g19190 (AtCXE1) | XP_016438323.1 | Solyc01g108520 | ES789969 (MdCXE2) | XP_019073806.1 |
|  | Prupe.8G120800 (PpCXE2) | At1g47480 (AtCXE2) | XP_016506603.1 | Solyc04g079190 | ES789964 (MdCXE3) | XP_010647769.1 |
|  | Prupe.8G121100 (PpCXE3) | At1g49640 (AtCXE3) | XP_016489385.1 | Solyc01g108580 (SlCXE5) | ES789968 (MdCXE5) | XP_002285071.1 |
|  | Prupe.8G120900 (PpCXE4) | At1g49650 (AtCXE4) | XP_016488327.1 | NP_001307232.1 (SlCXE1) | ES789979 (MdCXE6) | XP_002285067.1 |
|  | Prupe.8G121000 (PpCXE5) | At1g49660 (AtCXE5) | XP_016482184.1 | Solyc01g108570 (SlCXE4) | ES789974 (MdCXE7) | XP_002285064.2 |
|  | Prupe.8G121200 (PpCXE6) | At2g03550 (AtCXE7) | XP_016473518.1 | Solyc01g108540 (SlCXE2) | ES789984 (MdCXE8) | XP_002285042.1 |
|  | Prupe.8G121300 (PpCXE7) | At3g48690 (AtCXE12) | XP_016459170.1 | Solyc01g108560 (SlCXE3) | ES789970 (MdCXE10) | XP_002285041.1 |
|  | Prupe.8G121400 (PpCXE8) | At3g48700 (AtCXE13) | XP_016451317.1 | Solyc08g060930 |  | XP_002285045.1 |
|  | Prupe.8G121500 (PpCXE9) |  | XP_016446428.1 | Solyc05g051660 (SlASH1) |  | XP_010647771.1 |
|  | Prupe.8G121700 (PpCXE10) |  |  | Solyc05g051670 (SlASH2) |  | XP_002284587.1 |
|  | Prupe.8G122000 (PpCXE11) |  |  | Solyc01g108530 |  | XP_002284585.2 |
|  | Prupe.1G155100 (PpCXE12) |  |  |  |  |  |
|  | Prupe.1G155200 (PpCXE13) |  |  |  |  |  |
|  | Prupe.1G439300 (PpCXE14) |  |  |  |  |  |
|  | Prupe.1G480100 (PpCXE15) |  |  |  |  |  |

**Supplementary Table S1. Continued**

| Groups | Peach | Arabidopsis | Tobacco | Tomato | Apple | Grape |
| --- | --- | --- | --- | --- | --- | --- |
| Group 4 | Prupe.4G252600 (PpCXE22) | At3g05120 (AtCXE10) | XP_016507904.1 | Solyc10g054780 | ES789959 (MdCXE1) | XP_002268777.1 |
|  | Prupe.6G332800 (PpCXE23) | At3g63010 (AtCXE14) | XP_016472266.1 |  | ES790010 (MdCXE12) | XP_010644022.2 |
|  | Prupe.8G004100 (PpCXE24) | At5g23530 (AtCXE18) |  |  |  | XP_002268736.1 |
|  | Prupe.8G090400 (PpCXE25) | At5g27320 (AtCXE19) |  |  |  | XP_002268654.1 |
|  | Prupe.8G249800 (PpCXE26) |  |  |  |  | XP_002268861.1 |
|  |  |  |  |  |  | XP_002270210.1 |
|  |  |  |  |  |  | XP_010653056.1 |
| Group 5 | Prupe.6G149100 (PpCXE27) | At5g06570 (AtCXE15) | XP_016438916.1 | Solyc11g045460 | ES789966 (MdCXE9) | XP_002277507.1 |
|  | Prupe.7G089000 (PpCXE28) |  | XP_016482016.1 | Solyc11g071720 |  | XP_002268704.1 |
|  |  |  | XP_016470908.1 |  |  | XP_002267394.1 |
|  |  |  | XP_016444962.1 |  |  | XP_002266241.1 |
| Group 6 | Prupe.1G307700 (PpCXE29) | At1g68620 (AtCXE6) | XP_016440132.1 | Solyc05g009610 |  | XP_002279965.2 |
|  | Prupe.3G238500 (PpCXE30) | At5g16080 (AtCXE17) | XP_016487566.1 | Solyc02g085800 |  | XP_002278939.1 |
|  |  |  | XP_016474751.1 |  |  | XP_002285090.3 |
|  |  |  | XP_016441795.1 |  |  | XP_002285088.1 |
|  |  |  |  |  |  | XP_002285085.1 |
|  |  |  |  |  |  | XP_002285083.1 |
|  |  |  |  |  |  | XP_002285077.1 |
|  |  |  |  |  |  | XP_019075185.1 |
| Group 7 | Prupe.2G044700 (PpCXE31) |  | XP_016441243.1 | Solyc02g069800 (Lehsr203J) | EG631279 (MdCXE11) | XP_002278031.1 |
|  | Prupe.2G045000 (PpCXE32) |  | XP_016500077.1 |  | ES789965 (MdCXE13) | XP_002277680.1 |
|  | Prupe.2G045300 (PpCXE33) |  | XP_016474715.1 (Nthsr203J) |  |  | XP_002266969.3 |
|  |  |  | XP_016472904.1 |  |  |  |
|  |  |  | XP_016443660.1 |  |  |  |
|  |  |  | XP_016435333.1 |  |  |  |
|  |  |  | XP_016454131.1 |  |  |  |

**Supplementary Table S2.** MES members in peach, Arabidopsis, tobacco, tomato, apple and grape.

| Groups | Peach | Arabidopsis | Tobacco | Tomato | Apple | Grape |
| --- | --- | --- | --- | --- | --- | --- |
| Group 1 | Prupe.7G140900 (PpMES1) | At2g23620 (AtMES1) | XP_016440268.1 | Solyc03g070380 | XP_017182347.1 | XP_002263026.4 (VvMES1) |
|  | Prupe.7G142200 (PpMES2) | At2g23600 (AtMES2) | XP_016449127.1 | Solyc02g065240 | XP_008388737.1 | XP_003635143.3 (VvMES2) |
|  | Prupe.7G141000 (PpMES3) | At2g23610 (AtMES3) | XP_016456028.1 | Solyc02g065250 | XP_008339151.2 | XP_003635144.1 (VvMES3) |
|  | Prupe.7G141100 (PpMES4) | At2g23580 (AtMES4) | XP_016504460.1 | Solyc02g065260 | XP_008339163.1 | XP_002270043.1 (VvMES4) |
|  | Prupe.7G141200 (PpMES5) | At5g10300 (AtMES5) | XP_016504461.1 | Solyc01g108740 | XP_017183069.1 | XP_010646594.1 (VvMES5/VvMJE1) |
|  | Prupe.7G141300 (PpMES6) | At2g23550 (AtMES6) | XP_016472162.1 | Solyc01g108750 | XP_008354686.1 | XP_019073788.1 (VvMES6) |
|  | Prupe.7G141400 (PpMES7) | At2g23560 (AtMES7) | NP_001312442.1 (NtSABP2) | Solyc03g044790 | XP_008368952.2 | CBI18572.3 (VvMES7) |
|  | Prupe.7G141500 (PpMES8) | At2g23590 (AtMES8) | XP_016473030.1 | Solyc03g044740 | XP_017189612.1 | XP_019074651.1 (VvMES10) |
|  | Prupe.7G141700 (PpMES9) | At4g37150 (AtMES9) | XP_016449124.1 | Solyc02g065280 | XP_008354234.1 | XP_002284944.1 (VvMES11) |
|  | Prupe.7G142100 (PpMES10) | At3g50440 (AtMES10) | XP_016488275.1 | Solyc03g044820 | XP_008361380.1 | XP_002284928.2 (VvMES12) |
|  | Prupe.8G114700 (PpMES11) | At2g23570 (AtMES19) | XP_016500318.1 | XP_004234676.1 (SlMJE) | XP_008338218.1 | XP_002284907.3 (VvMES13) |
|  | Prupe.8G115000 (PpMES12) | At4g37140 (AtMES20) | XP_016449128.1 |  | XP_008344281.1 |  |
|  | Prupe.8G115300 (PpMES13) |  |  |  |  |  |
| Group 2 | Prupe.2G266100 (PpMES17) | At4g16690 (AtMES16) | XP_016435275.1 | Solyc06g048570 | XP_008376326.1 | XP_002281126.1 (VvMES14) |
|  | Prupe.6G040000 (PpMES18) | At3g10870 (AtMES17) | XP_016501016.1 | Solyc03g095550 | XP_008388372.1 |  |
|  |  | At5g58310 (AtMES18) | XP_016451705.1 |  |  |  |
|  |  |  | XP_016445522.1 |  |  |  |
| Group 3 | Prupe.1G550200 (PpMES14) | At3g29770 (AtMES11) | XP_016439051.1 | Solyc06g064870 | XP_008389409.1 | XP_002265339.1 (VvMES8) |
|  | Prupe.1G288600 (PpMES15) | At4g09900 (AtMES12) | XP_016435471.1 | Solyc02g089060 | XP_008339612.2 | XP_002282036.1 (VvMES9) |
|  | Prupe.3G252200 (PpMES16) | At1g26360 (AtMES13) | XP_016514376.1 | Solyc05g012180 | XP_008341788.1 | XP_002279659.2 (VvMES15) |
|  |  | At1g33990 (AtMES14) | XP_016497307.1 |  | XP_008352791.1 |  |
|  |  | At1g69240 (AtMES15) | XP_016495632.1 |  | XP_008378739.1 |  |
|  |  |  | XP_016493570.1 |  |  |  |
|  |  |  | XP_016485144.1 |  |  |  |
|  |  |  | XP_016484453.1 |  |  |  |
|  |  |  | XP_016477736.1 |  |  |  |
|  |  |  | XP_016468289.1 |  |  |  |
|  |  |  | XP_016457816.1 |  |  |  |
|  |  |  | XP_016453698.1 |  |  |  |

**Supplementary Table S3.** Primers used for qPCR analysis.

| Gene | Primers name | Sequence |
| --- | --- | --- |
| *PpTEF2* | qPCR-F | GGTGTGACGATGAAGAGTGATG |
|  | qPCR-R | TGAAGGAGAGGGAAGGTGAAAG |
| *PpCXE1* | qPCR-F | GAATGGTGCTGTGGAGGTCA |
|  | qPCR-R | GCGTTGGAGTGAGAAAGTGC |
| *PpCXE2* | qPCR-F | GAGGCCTGGCTCAACGATTA |
|  | qPCR-R | CGACACCCAATCTTCCCCAA |
| *PpCXE3* | qPCR-F | GCCCTGAAGAGTGGCTCAAT |
|  | qPCR-R | AGACCCTATCAGCCCCCAAT |
| *PpMES1* | qPCR-F | GTTCCAGCAGTGCAAACCAG |
|  | qPCR-R | TGTTCGGGCTTTGAGAGCAT |
| *PpMES2* | qPCR-F | AGGCTAGAGTCTGCTGGTCA |
|  | qPCR-R | GTGAAGGCTGGTGTGTGGTA |

**Supplementary Table S4.** Primers used for gene cloning and pET-6×HN vectors construction.

| Gene name | Primers name | Primer sequences |
| --- | --- | --- |
| *PpCXE1* | Clone-F | ATGAGCAACGAAGACTTAGCCCATG |
|  | Clone-R | ATCACAGCGTTGGAGTGAGAAAGTG |
|  | pET-N-F | AAGGCCTCTGTCGACATGAGCAACGAAGACTTAGC |
|  | pET-N-R | GCCAGAATTCGCAAGCTTTTAGTTCAAGAAAGAAACAA |
| *PpCXE2* | Clone-F | ATGGACCCAAAATCCTCCTCAAC |
|  | Clone-R | CGGCAACCTAGTCCTGATTTATG |
|  | pET-N-F | AAGGCCTCTGTCGACATGGACCCAAAATCCTCCTC |
|  | pET-N-R | GCCAGAATTCGCAAGCTTCTAGTCCTGATTTATGA |
| *PpCXE3* | Clone-F | AAGGAGATCGATGGCAACATGTTG |
|  | Clone-R | CGAAACAGAGAACCCAATTTCAGG |
|  | pET-N-F | AAGGCCTCTGTCGACATGGCAACATGTTGTGTCCT |
|  | pET-N-R | GCCAGAATTCGCAAGCTTTTATATATCCTGATTGATGA |
| *PpMES1* | Clone-F | ATGGAGAATCCGAAACACTTTG |
|  | Clone-R | GCATTACAATATTTTCTCAGCCAC |
|  | pET-N-F | AAGGCCTCTGTCGACATGGAGAATCCGAAACACTTTG |
|  | pET-N-R | AGAATTCGCAAGCTTGCATTACAATATTTTCTCAGCCAC |
| *PpMES2* | Clone-F | ATGGCAGCACCAAAAGAAGAG |
|  | Clone-R | CTTTTCAGCCGGATCCTAAC |
|  | pET-N-F | AAGGCCTCTGTCGACATGGCAGCACCAAAAGAAGAG |
|  | pET-N-R | AGAATTCGCAAGCTTTCAGCCGGATCCTAACATTGT |

**Supplementary Table S5.** Transcript levels of CXE and MES gene members during fruit development and different tissues used for Heatmap analysis in Figure 4. Data are presented as mean from three independent biological replicates of RNA-seq.

| Gene name | S1 | S2 | S3 | S4 | S5 | Leaf | Flower |
| --- | --- | --- | --- | --- | --- | --- | --- |
| *PpCXE1* | 3.56 | 14.51 | 18.38 | 106.09 | 125.32 | 11.45 | 105.62 |
| *PpCXE2* | 120.71 | 209.82 | 164.32 | 157.63 | 186.78 | 118.51 | 70.08 |
| *PpCXE3* | 20.12 | 57.53 | 109.49 | 74.23 | 56.59 | 22.84 | 71.94 |
| *PpCXE4* | 1.37 | 1.14 | 0.74 | 0.45 | 0.35 | 1.47 | 3.78 |
| *PpCXE5* | 7.05 | 5.68 | 5.07 | 1.76 | 0.48 | 0.73 | 5.38 |
| *PpCXE6* | 0.14 | 0.11 | 0.04 | 0.02 | 0.00 | 0.11 | 0.78 |
| *PpCXE7* | 0.00 | 0.00 | 0.00 | 0.00 | 0.00 | 0.04 | 67.65 |
| *PpCXE8* | 20.09 | 49.21 | 45.77 | 35.55 | 11.59 | 79.39 | 61.59 |
| *PpCXE9* | 10.03 | 29.01 | 28.99 | 12.92 | 3.49 | 102.98 | 37.33 |
| *PpCXE10* | 0.18 | 0.18 | 0.29 | 1.23 | 2.23 | 62.56 | 65.77 |
| *PpCXE11* | 0.04 | 0.04 | 0.00 | 0.00 | 0.00 | 0.29 | 5.21 |
| *PpCXE12* | 12.37 | 5.66 | 6.03 | 3.96 | 2.15 | 0.08 | 11.84 |
| *PpCXE13* | 3.48 | 8.13 | 1.12 | 3.12 | 2.37 | 58.61 | 146.86 |
| *PpCXE14* | 1.11 | 4.64 | 3.82 | 5.74 | 4.64 | 1.39 | 12.15 |
| *PpCXE15* | 0.26 | 0.30 | 0.26 | 0.27 | 0.40 | 3.16 | 1.45 |
| *PpCXE16* | 0.17 | 0.04 | 0.04 | 0.09 | 0.17 | 2.24 | 8.57 |
| *PpCXE17* | 33.96 | 39.37 | 31.42 | 40.25 | 36.98 | 86.41 | 21.59 |
| *PpCXE18* | 14.13 | 26.32 | 7.15 | 2.91 | 0.79 | 0.91 | 3.96 |
| *PpCXE19* | 0.03 | 0.04 | 0.34 | 0.29 | 0.14 | 0.01 | 0.93 |
| *PpCXE20* | 0.38 | 0.32 | 0.64 | 0.26 | 0.17 | 0.14 | 3.04 |
| *PpCXE21* | 0.07 | 1.70 | 0.89 | 2.12 | 1.01 | 1.38 | 46.41 |
| *PpCXE22* | 0.03 | 0.20 | 0.12 | 0.11 | 0.09 | 0.09 | 0.05 |
| *PpCXE23* | 51.90 | 44.56 | 55.97 | 23.59 | 27.48 | 14.52 | 138.32 |
| *PpCXE24* | 0.48 | 0.09 | 0.01 | 0.04 | 0.01 | 0.00 | 0.41 |
| *PpCXE25* | 4.10 | 2.78 | 1.82 | 5.67 | 11.81 | 2.71 | 16.07 |
| *PpCXE26* | 312.35 | 295.83 | 221.43 | 226.90 | 102.49 | 48.29 | 281.14 |
| *PpCXE27* | 0.63 | 0.52 | 1.94 | 4.63 | 7.28 | 4.09 | 0.63 |
| *PpCXE28* | 177.11 | 8.63 | 0.44 | 0.15 | 0.00 | 2.07 | 15.28 |
| *PpCXE29* | 2.06 | 0.14 | 0.06 | 0.09 | 0.03 | 0.73 | 6.36 |
| *PpCXE30* | 0.18 | 0.22 | 0.06 | 1.59 | 0.06 | 0.40 | 3.05 |
| *PpCXE31* | 0.00 | 0.00 | 0.00 | 0.00 | 0.00 | 0.00 | 0.00 |
| *PpCXE32* | 0.12 | 0.23 | 0.29 | 1.20 | 1.33 | 0.73 | 1.43 |
| *PpCXE33* | 0.56 | 0.61 | 0.85 | 0.03 | 0.00 | 12.43 | 18.94 |

**Supplementary Table S5.** Continued

| Gene name | S1 | S2 | S3 | S4 | S5 | Leaf | Flower |
| --- | --- | --- | --- | --- | --- | --- | --- |
| *PpMES1* | 4.26 | 2.95 | 13.19 | 8.95 | 13.18 | 0.82 | 3.12 |
| *PpMES2* | 6.49 | 6.23 | 5.70 | 0.68 | 0.18 | 0.12 | 1.29 |
| *PpMES3* | 0.02 | 0.32 | 1.14 | 2.08 | 3.58 | 39.67 | 130.31 |
| *PpMES4* | 0.02 | 0.00 | 0.06 | 0.33 | 0.06 | 0.01 | 1.97 |
| *PpMES5* | 0.01 | 0.00 | 0.30 | 0.10 | 0.01 | 0.30 | 26.51 |
| *PpMES6* | 7.75 | 0.03 | 0.32 | 0.52 | 0.16 | 94.00 | 46.02 |
| *PpMES7* | 2.71 | 0.00 | 0.03 | 0.08 | 0.05 | 37.97 | 14.68 |
| *PpMES8* | 0.77 | 2.16 | 0.18 | 0.27 | 0.05 | 224.56 | 20.82 |
| *PpMES9* | 0.00 | 0.00 | 0.01 | 0.00 | 0.00 | 0.00 | 0.00 |
| *PpMES10* | 0.11 | 0.16 | 0.09 | 0.06 | 0.08 | 0.05 | 0.01 |
| *PpMES11* | 0.06 | 0.06 | 0.17 | 0.06 | 0.04 | 0.05 | 0.03 |
| *PpMES12* | 0.03 | 0.14 | 0.02 | 0.00 | 0.03 | 0.02 | 0.36 |
| *PpMES13* | 0.00 | 0.00 | 0.00 | 0.00 | 0.00 | 0.06 | 0.04 |
| *PpMES14* | 15.19 | 11.56 | 12.19 | 8.06 | 7.03 | 5.86 | 9.07 |
| *PpMES15* | 0.41 | 1.83 | 1.02 | 0.39 | 0.26 | 2.18 | 13.45 |
| *PpMES16* | 8.16 | 9.80 | 6.76 | 10.21 | 10.86 | 16.26 | 5.34 |
| *PpMES17* | 0.26 | 0.44 | 9.33 | 22.29 | 21.25 | 3.24 | 6.73 |
| *PpMES18* | 82.52 | 41.76 | 21.18 | 10.81 | 8.92 | 0.39 | 14.02 |

**Supplementary Table S6.** Transcript levels of CXE and MES gene members in response to ethylene treatment used for Heatmap analysis in Figure 5. Data are presented as mean from three independent biological replicates of RNA-seq..

| Gene name | Control-12h | Ethylene-12h | Control-72h | Ethylene-72h |
| --- | --- | --- | --- | --- |
| *PpCXE1* | 14.22 | 79.96 | 52.39 | 95.97 |
| *PpCXE2* | 386.06 | 336.89 | 352.84 | 378.33 |
| *PpCXE3* | 126.01 | 171.88 | 154.44 | 190.08 |
| *PpCXE4* | 1.13 | 1.27 | 1.02 | 1.14 |
| *PpCXE5* | 7.26 | 3.10 | 3.32 | 4.43 |
| *PpCXE6* | 0.04 | 0.02 | 0.00 | 0.00 |
| *PpCXE7* | 0.00 | 0.00 | 0.00 | 0.00 |
| *PpCXE8* | 38.70 | 32.45 | 74.29 | 54.87 |
| *PpCXE9* | 11.79 | 8.34 | 22.75 | 22.05 |
| *PpCXE10* | 0.31 | 2.44 | 0.72 | 2.09 |
| *PpCXE11* | 0.00 | 0.00 | 0.00 | 0.00 |
| *PpCXE12* | 2.94 | 6.94 | 2.13 | 2.13 |
| *PpCXE13* | 0.11 | 4.79 | 0.21 | 0.29 |
| *PpCXE14* | 3.90 | 2.23 | 4.15 | 4.57 |
| *PpCXE15* | 0.27 | 0.33 | 0.41 | 0.50 |
| *PpCXE16* | 0.00 | 0.12 | 0.10 | 0.10 |
| *PpCXE17* | 55.56 | 62.03 | 52.07 | 52.59 |
| *PpCXE18* | 11.95 | 3.85 | 4.10 | 3.79 |
| *PpCXE19* | 0.51 | 0.40 | 0.28 | 0.38 |
| *PpCXE20* | 0.76 | 0.58 | 0.75 | 0.66 |
| *PpCXE21* | 1.09 | 1.56 | 1.01 | 0.88 |
| *PpCXE22* | 0.25 | 0.06 | 0.10 | 0.15 |
| *PpCXE23* | 34.79 | 14.71 | 19.90 | 25.94 |
| *PpCXE24* | 0.05 | 0.05 | 0.15 | 0.13 |
| *PpCXE25* | 7.22 | 7.98 | 9.55 | 12.43 |
| *PpCXE26* | 139.22 | 171.66 | 98.51 | 143.54 |
| *PpCXE27* | 1.45 | 2.45 | 6.13 | 8.04 |
| *PpCXE28* | 0.91 | 0.96 | 2.70 | 0.91 |
| *PpCXE29* | 0.00 | 0.01 | 0.03 | 0.00 |
| *PpCXE30* | 0.03 | 0.67 | 0.00 | 0.10 |
| *PpCXE31* | 0.00 | 0.00 | 0.00 | 0.00 |
| *PpCXE32* | 0.01 | 0.12 | 0.06 | 0.15 |
| *PpCXE33* | 0.04 | 0.35 | 0.16 | 0.02 |

**Supplementary Table S6.** Continued

| Gene name | Control-12h | Ethylene-12h | Control-72h | Ethylene-72h |
| --- | --- | --- | --- | --- |
| *PpMES1* | 13.43 | 11.95 | 14.84 | 19.68 |
| *PpMES2* | 15.32 | 2.83 | 11.54 | 5.62 |
| *PpMES3* | 0.09 | 1.83 | 0.58 | 1.15 |
| *PpMES4* | 0.00 | 0.00 | 0.00 | 0.00 |
| *PpMES5* | 0.00 | 0.00 | 0.00 | 0.00 |
| *PpMES6* | 0.00 | 1.00 | 0.10 | 0.01 |
| *PpMES7* | 0.00 | 0.07 | 0.00 | 0.03 |
| *PpMES8* | 0.05 | 0.19 | 0.29 | 0.11 |
| *PpMES9* | 0.00 | 0.01 | 0.00 | 0.00 |
| *PpMES10* | 0.90 | 0.09 | 0.33 | 0.27 |
| *PpMES11* | 0.31 | 0.11 | 0.23 | 0.05 |
| *PpMES12* | 0.11 | 0.08 | 0.00 | 0.00 |
| *PpMES13* | 0.00 | 0.00 | 0.00 | 0.00 |
| *PpMES14* | 25.04 | 27.56 | 24.24 | 24.01 |
| *PpMES15* | 0.92 | 0.67 | 0.66 | 0.70 |
| *PpMES16* | 8.55 | 7.80 | 10.36 | 12.48 |
| *PpMES17* | 4.55 | 12.58 | 8.61 | 18.89 |
| *PpMES18* | 63.85 | 28.00 | 43.39 | 29.64 |

**Supplementary Table S7.**  Transcript levels of CXE and MES gene members in response to MeJA treatment used for Heatmap analysis in Figure 6. Data are presented as mean from three independent biological replicates of RNA-seq.

| Gene name | Control-1d | MeJA-1d | Control-3d | MeJA-3d |
| --- | --- | --- | --- | --- |
| *PpCXE1* | 64.28 | 181.40 | 121.58 | 106.22 |
| *PpCXE2* | 226.76 | 265.05 | 209.58 | 175.44 |
| *PpCXE3* | 61.36 | 80.58 | 76.29 | 59.35 |
| *PpCXE4* | 0.37 | 0.77 | 0.57 | 0.47 |
| *PpCXE5* | 2.80 | 2.59 | 2.15 | 1.63 |
| *PpCXE6* | 0.00 | 0.03 | 0.00 | 0.03 |
| *PpCXE7* | 0.05 | 0.04 | 0.04 | 0.00 |
| *PpCXE8* | 15.01 | 31.51 | 14.69 | 19.38 |
| *PpCXE9* | 3.92 | 7.45 | 3.68 | 7.02 |
| *PpCXE10* | 3.86 | 25.74 | 9.63 | 21.26 |
| *PpCXE11* | 0.01 | 0.03 | 0.07 | 0.02 |
| *PpCXE12* | 2.03 | 5.06 | 1.34 | 2.35 |
| *PpCXE13* | 1.95 | 7.20 | 1.50 | 3.00 |
| *PpCXE14* | 1.87 | 1.77 | 1.57 | 1.71 |
| *PpCXE15* | 0.15 | 0.11 | 0.06 | 0.06 |
| *PpCXE16* | 0.28 | 0.15 | 0.21 | 0.47 |
| *PpCXE17* | 40.33 | 39.21 | 37.89 | 40.23 |
| *PpCXE18* | 1.12 | 0.63 | 0.32 | 0.24 |
| *PpCXE19* | 0.11 | 0.03 | 0.12 | 0.09 |
| *PpCXE20* | 0.34 | 0.29 | 0.28 | 0.29 |
| *PpCXE21* | 0.79 | 1.29 | 0.85 | 2.16 |
| *PpCXE22* | 0.02 | 0.02 | 0.06 | 0.10 |
| *PpCXE23* | 12.75 | 10.52 | 16.78 | 9.51 |
| *PpCXE24* | 0.14 | 0.21 | 0.07 | 0.05 |
| *PpCXE25* | 4.70 | 3.62 | 5.30 | 7.16 |
| *PpCXE26* | 25.94 | 18.44 | 26.23 | 17.41 |
| *PpCXE27* | 0.61 | 0.33 | 0.75 | 1.07 |
| *PpCXE28* | 0.02 | 0.08 | 0.02 | 0.02 |
| *PpCXE29* | 0.06 | 0.09 | 0.12 | 0.09 |
| *PpCXE30* | 0.11 | 0.44 | 0.04 | 0.19 |
| *PpCXE31* | 0.00 | 0.02 | 0.00 | 0.01 |
| *PpCXE32* | 0.40 | 0.03 | 0.27 | 0.51 |
| *PpCXE33* | 0.64 | 5.03 | 0.25 | 1.48 |

**Supplementary Table S7.** Continued

| Gene name | Control-1d | MeJA-1d | Control-3d | MeJA-3d |
| --- | --- | --- | --- | --- |
| *PpMES1* | 25.36 | 24.02 | 22.42 | 32.12 |
| *PpMES2* | 1.26 | 1.41 | 0.74 | 0.49 |
| *PpMES3* | 4.41 | 35.11 | 13.31 | 32.62 |
| *PpMES4* | 0.09 | 0.12 | 0.54 | 1.99 |
| *PpMES5* | 0.21 | 0.01 | 0.03 | 0.22 |
| *PpMES6* | 0.44 | 0.85 | 0.11 | 1.41 |
| *PpMES7* | 0.09 | 0.19 | 0.07 | 0.29 |
| *PpMES8* | 0.04 | 0.09 | 0.10 | 0.25 |
| *PpMES9* | 0.00 | 0.00 | 0.00 | 0.00 |
| *PpMES10* | 0.18 | 0.18 | 0.18 | 0.18 |
| *PpMES11* | 0.43 | 0.58 | 0.21 | 0.23 |
| *PpMES12* | 0.02 | 0.11 | 0.02 | 0.02 |
| *PpMES13* | 0.00 | 0.00 | 0.00 | 0.00 |
| *PpMES14* | 10.75 | 15.13 | 11.01 | 12.08 |
| *PpMES15* | 0.28 | 0.73 | 0.33 | 0.54 |
| *PpMES16* | 6.23 | 5.55 | 6.85 | 8.77 |
| *PpMES17* | 7.64 | 9.25 | 6.50 | 4.47 |
| *PpMES18* | 14.82 | 17.10 | 14.39 | 9.71 |

**Supplementary Table S8.**  Transcript levels of CXE and MES gene members in response to UV-B treatment used for Heatmap analysis in Figure 7. Data are presented as mean from three independent biological replicates of RNA-seq.

| Gene name | Control-6h | UV-B-6h | Control-48h | UV-B-48h |
| --- | --- | --- | --- | --- |
| *PpCXE1* | 37.66 | 49.45 | 85.74 | 580.24 |
| *PpCXE2* | 299.25 | 320.48 | 210.66 | 255.60 |
| *PpCXE4* | 0.65 | 1.45 | 0.33 | 1.28 |
| *PpCXE5* | 1.93 | 5.24 | 1.78 | 2.85 |
| *PpCXE3* | 112.40 | 144.53 | 98.79 | 118.91 |
| *PpCXE6* | 0.00 | 0.10 | 0.00 | 0.00 |
| *PpCXE7* | 0.00 | 0.03 | 0.00 | 0.00 |
| *PpCXE8* | 13.09 | 67.02 | 8.39 | 186.73 |
| *PpCXE9* | 5.07 | 16.57 | 3.62 | 136.33 |
| *PpCXE10* | 4.01 | 27.31 | 11.73 | 1071.77 |
| *PpCXE11* | 0.00 | 0.00 | 0.00 | 0.00 |
| *PpCXE12* | 0.94 | 33.33 | 0.95 | 24.31 |
| *PpCXE13* | 3.17 | 77.13 | 2.43 | 90.83 |
| *PpCXE14* | 12.03 | 46.87 | 9.36 | 23.31 |
| *PpCXE15* | 0.21 | 0.17 | 0.62 | 0.54 |
| *PpCXE16* | 0.73 | 19.84 | 0.62 | 17.34 |
| *PpCXE17* | 32.67 | 35.10 | 30.70 | 47.42 |
| *PpCXE18* | 9.50 | 4.59 | 4.70 | 2.04 |
| *PpCXE19* | 0.38 | 1.54 | 0.20 | 1.21 |
| *PpCXE20* | 0.21 | 1.00 | 0.31 | 0.49 |
| *PpCXE21* | 5.75 | 91.60 | 3.08 | 52.55 |
| *PpCXE22* | 4.21 | 0.47 | 4.34 | 0.19 |
| *PpCXE23* | 5.70 | 7.52 | 7.78 | 5.45 |
| *PpCXE24* | 0.00 | 0.03 | 0.02 | 0.00 |
| *PpCXE25* | 8.98 | 14.19 | 10.51 | 8.55 |
| *PpCXE26* | 62.50 | 107.93 | 87.05 | 246.14 |
| *PpCXE27* | 0.71 | 1.12 | 1.77 | 4.37 |
| *PpCXE28* | 0.00 | 0.32 | 0.00 | 0.00 |
| *PpCXE29* | 0.00 | 0.00 | 0.00 | 0.04 |
| *PpCXE30* | 0.00 | 0.00 | 0.03 | 0.04 |
| *PpCXE31* | 0.00 | 0.00 | 0.03 | 0.00 |
| *PpCXE32* | 0.62 | 2.41 | 0.31 | 1.92 |
| *PpCXE33* | 0.35 | 22.72 | 0.19 | 20.22 |

**Supplementary Table S8.** Continued

| Gene name | Control-6h | UV-B-6h | Control-48h | UV-B-48h |
| --- | --- | --- | --- | --- |
| *PpMES1* | 24.24 | 15.53 | 16.95 | 18.01 |
| *PpMES2* | 2.67 | 2.81 | 1.55 | 0.71 |
| *PpMES3* | 1.01 | 13.79 | 3.81 | 393.54 |
| *PpMES4* | 2.19 | 7.19 | 5.23 | 397.46 |
| *PpMES5* | 1.14 | 16.07 | 0.74 | 172.38 |
| *PpMES6* | 2.85 | 9.36 | 11.41 | 1009.79 |
| *PpMES7* | 0.80 | 1.80 | 2.65 | 112.28 |
| *PpMES8* | 0.08 | 3.11 | 0.43 | 47.86 |
| *PpMES9* | 0.00 | 0.12 | 0.04 | 2.26 |
| *PpMES10* | 0.16 | 0.19 | 0.31 | 0.10 |
| *PpMES11* | 0.16 | 0.19 | 0.30 | 0.12 |
| *PpMES12* | 0.13 | 1.38 | 0.28 | 4.62 |
| *PpMES13* | 0.00 | 0.00 | 0.00 | 0.13 |
| *PpMES14* | 15.51 | 13.29 | 15.26 | 9.22 |
| *PpMES15* | 3.29 | 18.58 | 1.23 | 6.27 |
| *PpMES16* | 22.51 | 6.78 | 22.98 | 11.38 |
| *PpMES17* | 1.24 | 8.01 | 2.71 | 13.16 |
| *PpMES18* | 40.16 | 19.87 | 15.49 | 4.11 |

**Supplementary Table S9.** Analysis of cis-acting CXE and MES gene promoter regulatory elements in peach.

| Classes | Groups | Gene | Light | ABA | MeJA | SA | Auxin | Defense | Wound | Low temperature | MYB binding site | MYC binding site |
| --- | --- | --- | --- | --- | --- | --- | --- | --- | --- | --- | --- | --- |
| CXEs | Group 1 | PpCXE16 | 8 | 2 | 1 | 0 | 0 | 0 | 0 | 3 | 6 | 1 |
|  |  | PpCXE17 | 11 | 2 | 1 | 4 | 0 | 0 | 1 | 0 | 7 | 10 |
|  | Group 2 | PpCXE18 | 8 | 1 | 1 | 1 | 0 | 0 | 0 | 1 | 5 | 2 |
|  |  | PpCXE19 | 9 | 3 | 0 | 0 | 2 | 0 | 1 | 0 | 6 | 4 |
|  |  | PpCXE20 | 7 | 4 | 7 | 0 | 3 | 0 | 2 | 3 | 8 | 1 |
|  |  | PpCXE21 | 10 | 3 | 4 | 1 | 1 | 1 | 0 | 5 | 5 | 4 |
|  | Group 3 | PpCXE1 | 18 | 4 | 4 | 3 | 1 | 0 | 0 | 2 | 5 | 10 |
|  |  | PpCXE2 | 8 | 4 | 1 | 0 | 2 | 0 | 0 | 2 | 16 | 2 |
|  |  | PpCXE3 | 8 | 3 | 2 | 2 | 0 | 0 | 1 | 0 | 7 | 8 |
|  |  | PpCXE4 | 4 | 5 | 1 | 0 | 3 | 0 | 0 | 4 | 8 | 4 |
|  |  | PpCXE5 | 9 | 4 | 1 | 0 | 1 | 0 | 3 | 0 | 6 | 6 |
|  |  | PpCXE6 | 6 | 2 | 3 | 1 | 1 | 1 | 1 | 2 | 7 | 2 |
|  |  | PpCXE7 | 11 | 2 | 2 | 1 | 2 | 1 | 0 | 0 | 7 | 3 |
|  |  | PpCXE8 | 7 | 2 | 0 | 1 | 1 | 1 | 0 | 0 | 2 | 8 |
|  |  | PpCXE9 | 5 | 1 | 1 | 1 | 0 | 1 | 0 | 1 | 10 | 8 |
|  |  | PpCXE10 | 13 | 4 | 1 | 2 | 1 | 1 | 1 | 0 | 5 | 6 |
|  |  | PpCXE11 | 11 | 2 | 3 | 2 | 1 | 0 | 1 | 0 | 6 | 4 |
|  |  | PpCXE12 | 7 | 2 | 1 | 0 | 0 | 0 | 2 | 2 | 8 | 3 |
|  |  | PpCXE13 | 10 | 0 | 0 | 2 | 0 | 0 | 1 | 2 | 6 | 4 |
|  |  | PpCXE14 | 9 | 3 | 0 | 1 | 1 | 1 | 2 | 0 | 6 | 4 |
|  |  | PpCXE15 | 6 | 0 | 1 | 0 | 0 | 1 | 0 | 0 | 7 | 4 |
|  | Group 4 | PpCXE22 | 11 | 8 | 3 | 0 | 1 | 0 | 0 | 1 | 6 | 5 |
|  |  | PpCXE23 | 20 | 7 | 5 | 0 | 1 | 2 | 2 | 2 | 8 | 4 |
|  |  | PpCXE24 | 13 | 4 | 2 | 0 | 2 | 1 | 1 | 2 | 9 | 4 |
|  |  | PpCXE25 | 10 | 3 | 0 | 0 | 3 | 0 | 0 | 0 | 6 | 4 |
|  |  | PpCXE26 | 7 | 1 | 1 | 4 | 0 | 0 | 1 | 0 | 7 | 4 |
|  | Group 5 | PpCXE27 | 11 | 0 | 0 | 3 | 0 | 1 | 1 | 0 | 5 | 3 |
|  |  | PpCXE28 | 13 | 1 | 0 | 1 | 2 | 0 | 0 | 0 | 4 | 0 |
|  | Group 6 | PpCXE29 | 9 | 3 | 2 | 1 | 1 | 1 | 1 | 0 | 6 | 7 |
|  |  | PpCXE30 | 9 | 4 | 2 | 3 | 0 | 0 | 0 | 0 | 3 | 6 |
|  | Group 7 | PpCXE31 | 12 | 1 | 0 | 2 | 0 | 0 | 0 | 1 | 4 | 6 |
|  |  | PpCXE32 | 12 | 3 | 4 | 2 | 1 | 0 | 0 | 0 | 5 | 1 |
|  |  | PpCXE33 | 11 | 3 | 0 | 1 | 1 | 0 | 1 | 2 | 6 | 1 |
| MESs | Group 1 | PpMES1 | 14 | 4 | 1 | 0 | 0 | 1 | 1 | 0 | 8 | 0 |
|  |  | PpMES2 | 7 | 2 | 1 | 2 | 1 | 1 | 0 | 0 | 5 | 7 |
|  |  | PpMES3 | 10 | 4 | 4 | 0 | 3 | 0 | 0 | 1 | 6 | 10 |
|  |  | PpMES4 | 10 | 5 | 1 | 0 | 3 | 1 | 0 | 0 | 2 | 8 |
|  |  | PpMES5 | 9 | 3 | 1 | 0 | 0 | 0 | 1 | 0 | 3 | 8 |
|  |  | PpMES6 | 11 | 3 | 1 | 1 | 0 | 0 | 0 | 0 | 0 | 6 |
|  |  | PpMES7 | 5 | 1 | 1 | 0 | 0 | 0 | 1 | 0 | 4 | 4 |
|  |  | PpMES8 | 6 | 3 | 1 | 2 | 0 | 0 | 0 | 0 | 5 | 6 |
|  |  | PpMES9 | 7 | 2 | 2 | 2 | 1 | 0 | 0 | 0 | 5 | 3 |
|  |  | PpMES10 | 13 | 3 | 0 | 0 | 2 | 0 | 2 | 0 | 6 | 6 |
|  |  | PpMES11 | 8 | 0 | 0 | 2 | 0 | 2 | 2 | 0 | 8 | 5 |
|  |  | PpMES12 | 11 | 1 | 1 | 2 | 0 | 1 | 0 | 0 | 12 | 8 |
|  |  | PpMES13 | 9 | 0 | 2 | 2 | 1 | 1 | 2 | 0 | 8 | 4 |
|  | Group 2 | PpMES17 | 5 | 1 | 2 | 2 | 2 | 0 | 2 | 0 | 6 | 8 |
|  |  | PpMES18 | 11 | 0 | 2 | 3 | 0 | 0 | 3 | 0 | 5 | 8 |
|  | Group 3 | PpMES14 | 12 | 3 | 2 | 1 | 0 | 2 | 1 | 2 | 8 | 6 |
|  |  | PpMES15 | 9 | 3 | 1 | 3 | 2 | 0 | 1 | 1 | 7 | 7 |
|  |  | PpMES16 | 13 | 2 | 1 | 2 | 1 | 0 | 0 | 1 | 4 | 7 |

**
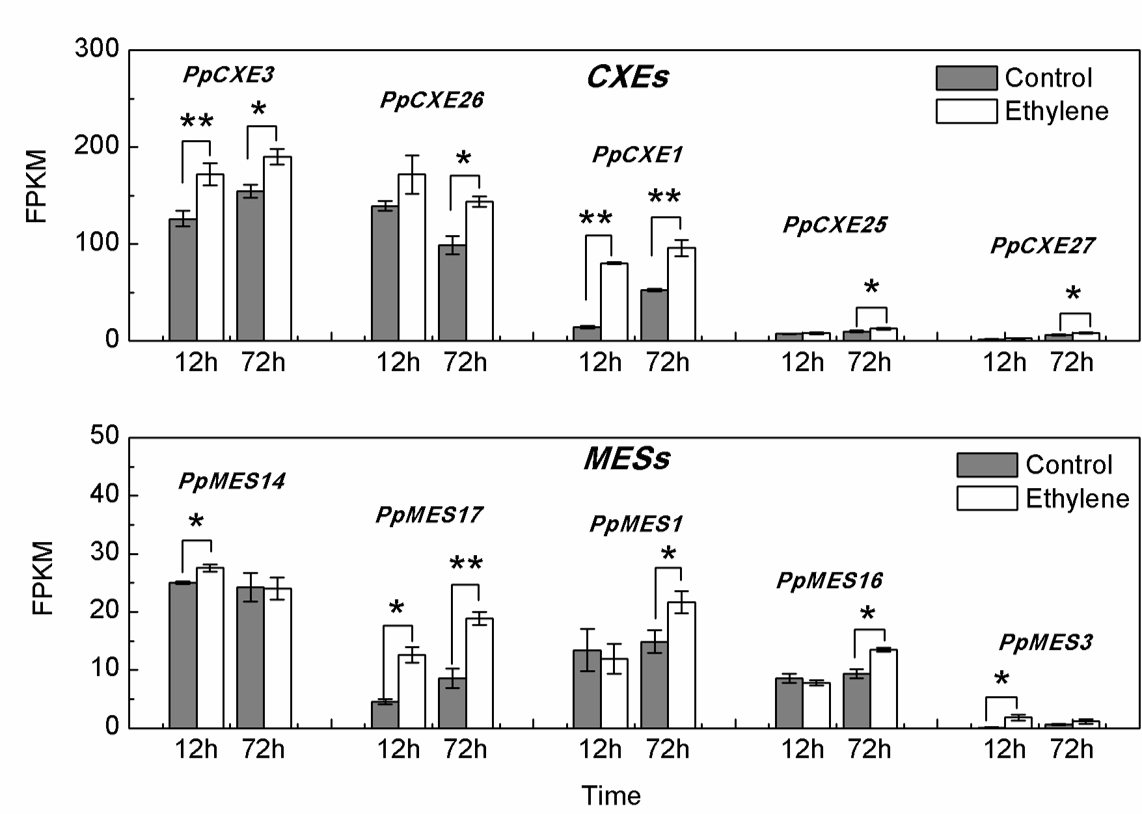
**

**Supplementary Figure S1.** Transcript levels of the top 5 most highly expressed CXE and MES genes up-regulated by ethylene. 12 h and 72 h represent hours after peach fruit harvest. Error bars represent SE (n=3). * significantly different (*P<0.05*), ** significantly different (*P<0.01*).


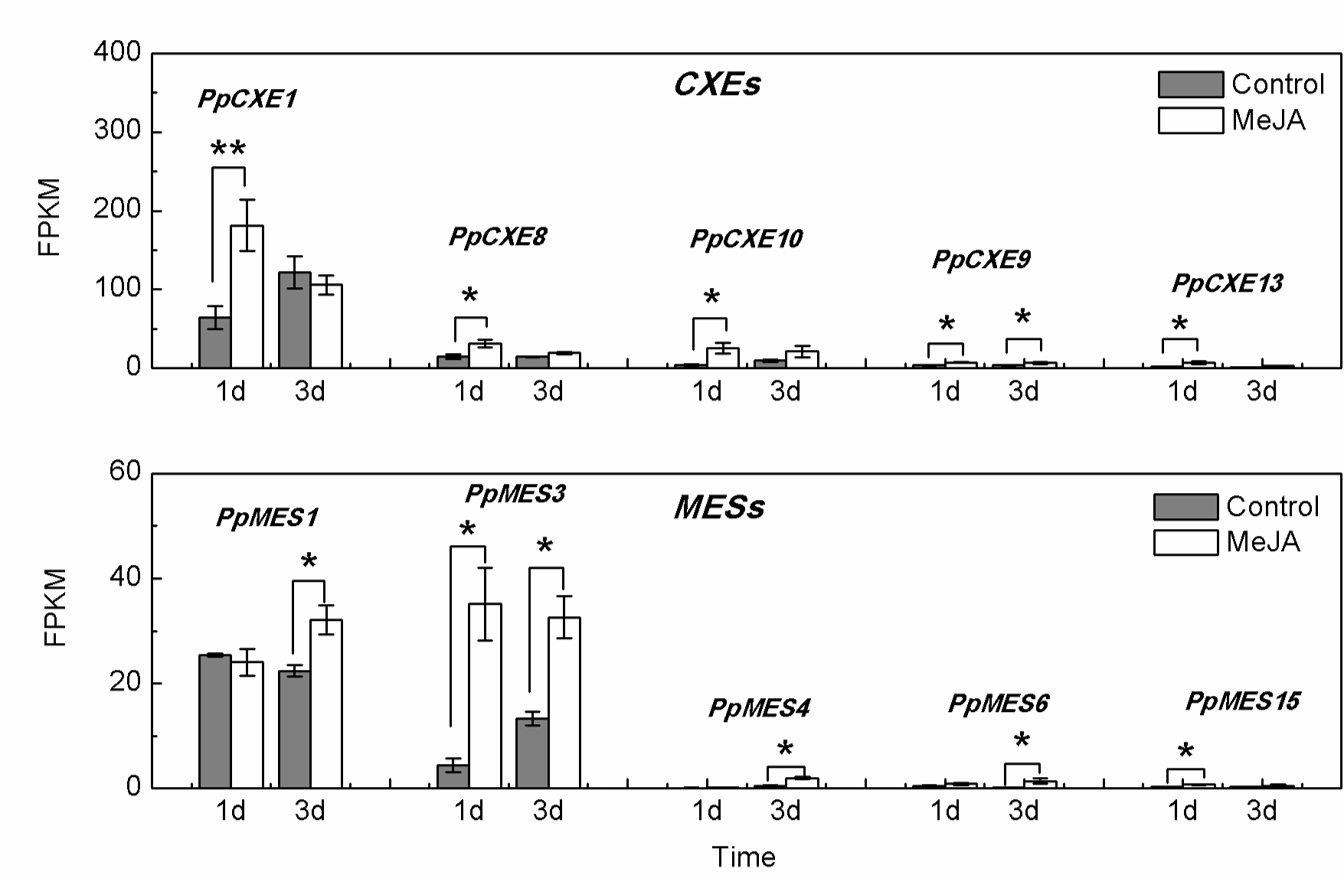


**Supplementary Figure S2.** Transcript levels of the top 5 most highly expressed CXE and MES genes up-regulated by MeJA. 1 d and 3 d represent days after MeJA treatment. Error bars represent SE (n=3). * significantly different (*P<0.05*), ** significantly different (*P<0.01*).

**
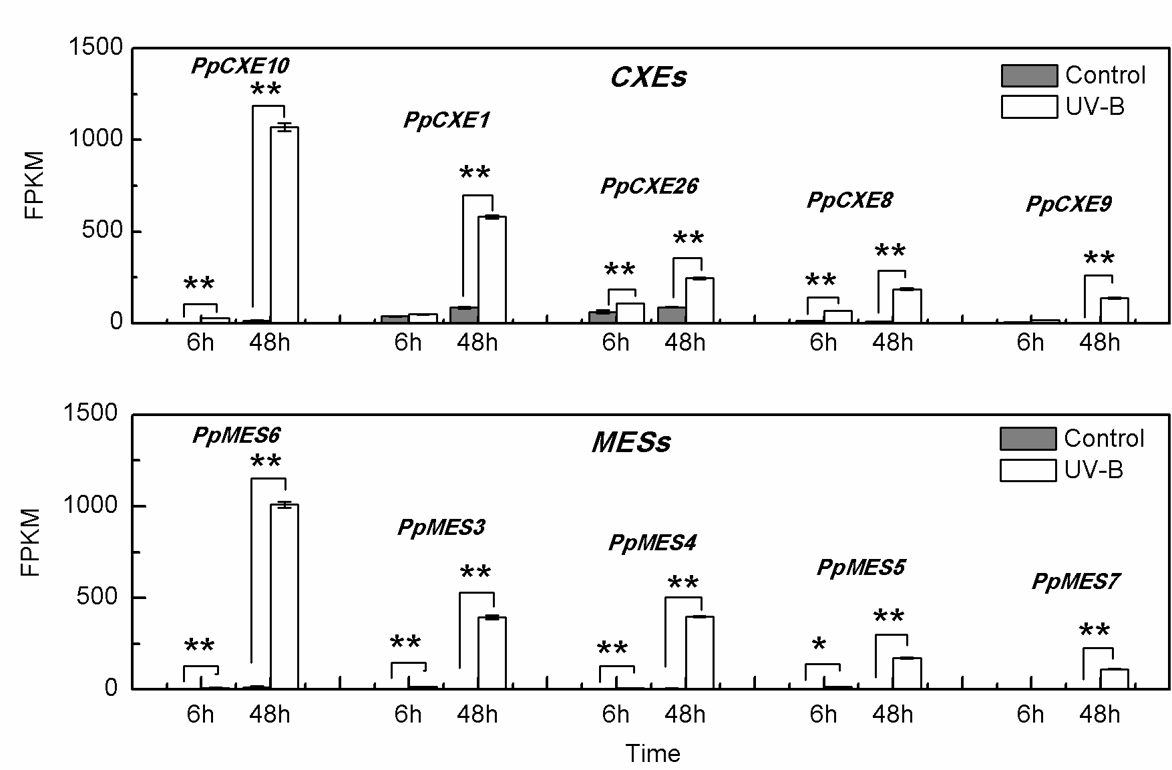
**

**Supplementary Figure S3.** Transcript levels of the top 5 most highly expressed CXE and MES genes up-regulated by UV-B. 6 h and 48 h represent hours after peach fruit treated with UV-B. Error bars represent SE (n=3). * significantly different (*P<0.05*), ** significantly different (*P<0.01*).





**Supplementary Figure S4.** Transcript levels of CXE and MES genes analyzed by RNA-seq and qPCR during fruit ripening. Error bars represent SE (n=3).





**Supplementary Figure S5.** Measurement by qPCR of transcript levels of CXE and MES genes in response to ethylene treatment. Error bars represent SE (n=3). * significantly different (*P<0.05*), ** significantly different (*P<0.01*).





**Supplementary Figure S6.** Measurement by qPCR of transcript levels of CXE and MES genes in response to MeJA treatment. Error bars represent SE (n=3). * significantly different (*P<0.05*), ** significantly different (*P<0.01*).





**Supplementary Figure S7.** Measurement by qPCR of transcript levels of CXE and MES genes in response to UV-B treatment. Error bars represent SE (n=3). * significantly different (*P<0.05*), ** significantly different (*P<0.01*).


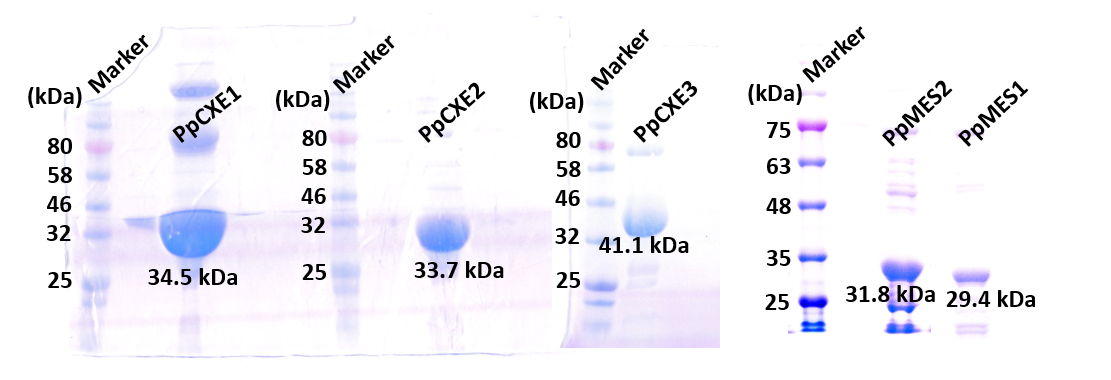


**Supplementary Figure S8**. SDS-PAGE analysis of purified CXE and MES recombinant proteins. PpCXE1, PpCXE2, PpCXE3, PpMES1 and PpMES2 represent the recombinant proteins purified from *E.coli*. Positions of the size marker proteins have been indicated. The numbers below the band indicate the molecular weight of the proteins.


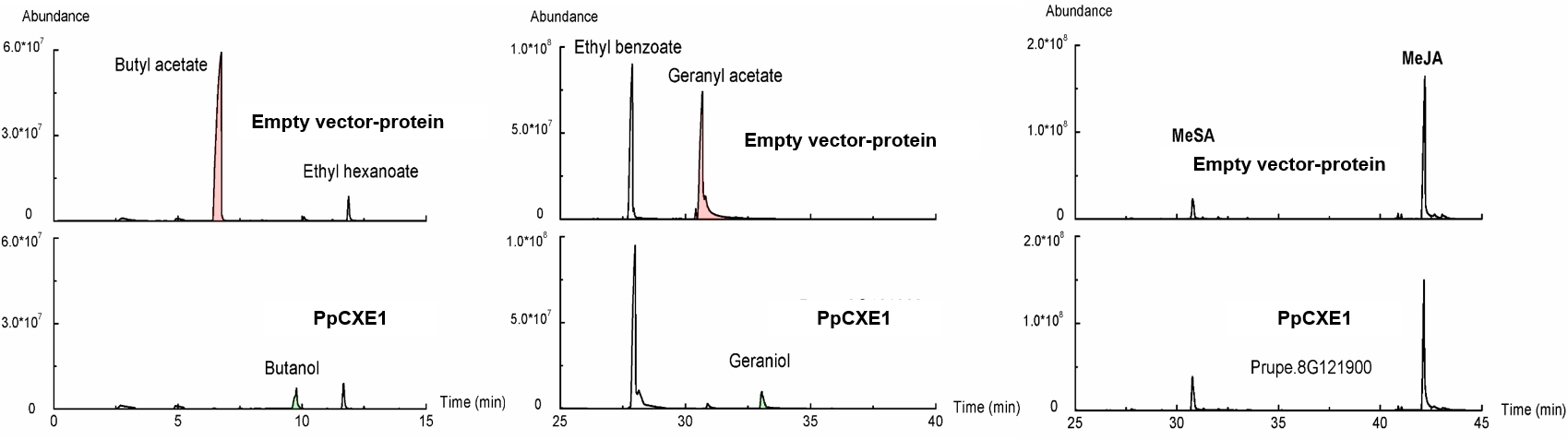


**Supplementary Figure S9.** Enzymatic activity analysis of PpCXE1 recombinant protein. Different esters were used as substrates, including butyl acetate, ethyl hexanoate, ethyl benzoate, geranyl acetate, MeSA and MeJA.


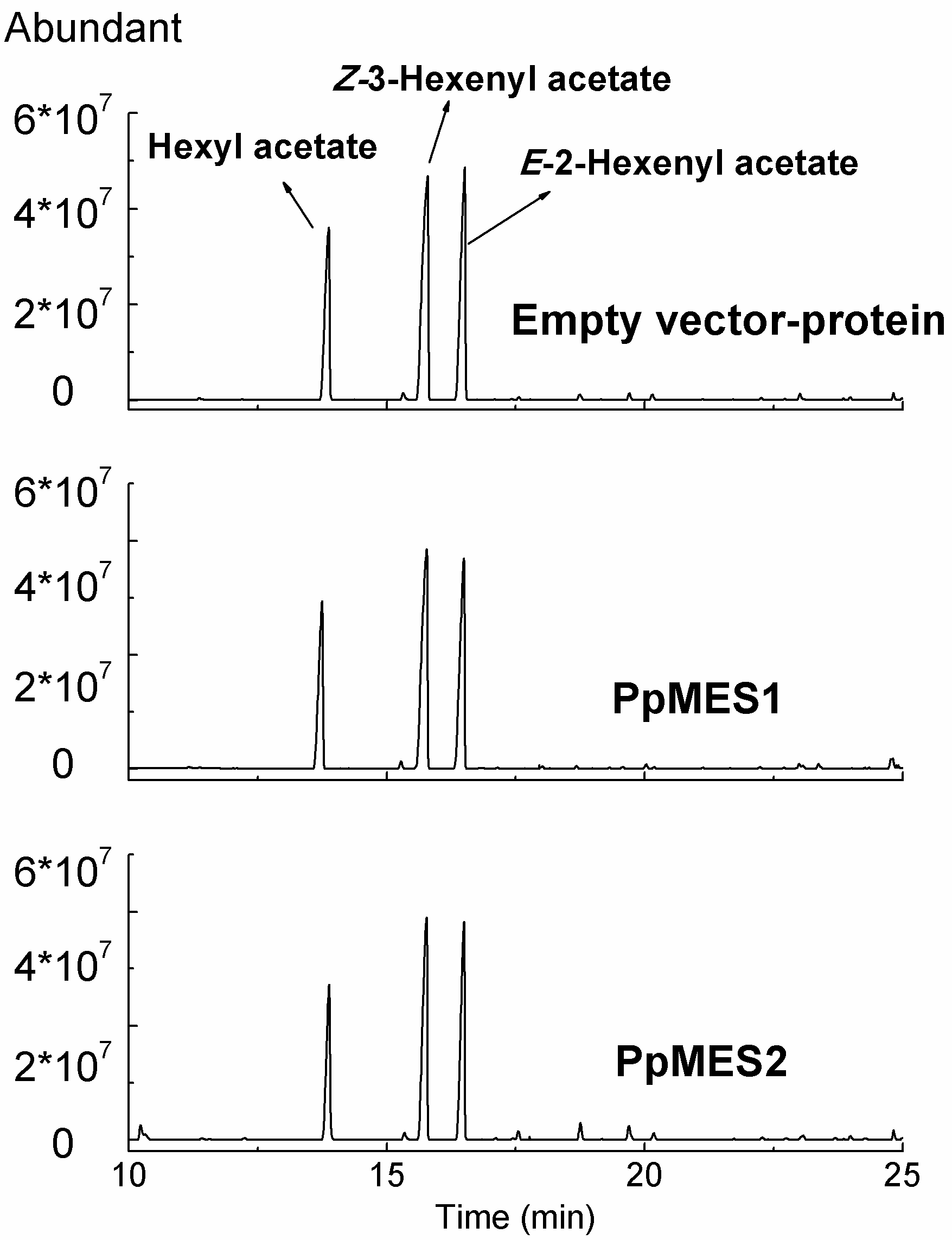


**Supplementary Figure S10.** Enzymatic activity analysis of PpMES1 and PpMES2 recombinant protein. Different esters were used as substrates, including hexyl acetate, *Z-*3-hexenyl acetate and *E*-2-hexenyl acetate.
